# Stable H-bond networks are crucial for selective CLK1 inhibition: a computational perspective

**DOI:** 10.3389/fchem.2025.1582515

**Published:** 2025-06-17

**Authors:** Yuzhou Huang, Baichun Hu, Haihan Liu, Jian Wang, Na Duan

**Affiliations:** ^1^ Department of Cardiology, The People’s Hospital of China Medical University, The People’s Hospital of Liaoning Province, Shenyang, China; ^2^ Key Laboratory of Structure-Based Drug Design & Discovery of Ministry of Education, Shenyang Pharmaceutical University, Shenyang, China

**Keywords:** CLK1, CLK3, selective inhibitor, molecular docking, molecular dynamics simulation

## Abstract

Studying the selectivity mechanism of inhibitors towards highly similar isoforms is an important task in the development of new drugs, which are designed to avoid the undesired side effects *in vivo*. CDC-like kinase isoforms (CLKs) are serine/threonine protein kinases that are involved in the phosphorylation of mRNA spliceosomes leading to the regulation of gene expression. The CLK isoforms are expressed in most human tissues and cells, but the expression levels of each isoform vary in different cells. Typically, CLK3 is expressed in male testes and sperm, by contrast, as a potential cancer treatment target, the expression level of CLK1 in testicular tissue is significantly lower than other isoforms. These differences in the tissue distribution of CLK1 and CLK3 suggest that the development of selective CLK1 inhibitors to avoid potential side effects. Here, our study is designed to reveal the selectivity mechanism of CLK1 inhibition from a computational perspective. In this study, the binding modes of known selective inhibitors towards CLK1/3 are discussed by computational methods such as protein comparison, molecular docking, binding free energy calculation, molecular dynamics simulations, alanine mutagenesis simulations, and quantum mechanical calculation. The simulations reveal selective key roles involved in CLK1/3 binding, including protein-ligand interactions, mutations, and conformational differences in key amino acid residues. This study will contribute to analyze the selectivity mechanism of CLKs inhibitors and bring insight into the development of novel selective inhibitor drugs.

## Introduction

Protein kinases (PK) catalyze the process of protein phosphorylation which play a key role in cellular signaling and gene expression (Roskoski, 2015) through transferring γ-phosphate group from nucleoside triphosphate to hydroxyl group of serine, threonine, or tyrosine residue of the regulated protein, thus causing conformational changes and leading to “on-off” protein function or greatly increasing or decreasing the enzymatic activity of the protein (Modi and Dunbrack, 2019; ter Haar et al., 2004). Phosphorylation of proteins can lead to changes in cellular signaling pathways that regulate key cell cycle processes such as cell metabolism, growth, proliferation, differentiation, and apoptosis. Therefore, various protein kinases as the core of signal transduction pathways have long been the most important and efficient drug targets.

Mutations and dysregulation of protein kinases are often associated with the development of cancer, and nowadays a variety of highly targeted drugs have been developed (Cohen, 2002; Deng et al., 2019; Fleuren et al., 2016). Protein kinases are capable of driving selective splicing and constitutive splicing processes of mRNAs, leading to complexity and diversity of gene transcription (Corkery et al., 2015). The splicing process is mainly driven by protein kinases that phosphorylate splicing factors, which are usually located at the central part of the spliceosome and are called serine/arginine-rich (SR) proteins. Activation and phosphorylation of SR proteins can be carried out by SR protein kinases (SRPKs), cAMP-dependent protein kinase (PKA), and the family of CDC-like kinases (CLKs) (Ding et al., 2012; Shi et al., 2011). CLKs are evolutionarily conserved dual-specific kinases that catalyze the phosphorylation of SR proteins and splicing factors 1–22 (SRSF1-12), thereby regulating the protein conformation of the spliceosomes and causing changes in the expression of genetic information (Aubol et al., 2016; Hochberg-Laufer et al., 2019; Moyano et al., 2020).

CLKs are considered promising chemo-therapeutic targets which attract increasing attention in the field of drug development. For example, CLK inhibitor SM08502 has recently entered clinical trials for the treatment of advanced solid tumors (Tam et al., 2020), and another inhibitor CX-4945 has been used for antitumor therapy against glioblastoma (Park et al., 2016). Meanwhile, increased expression of CLK1 inhibits HIV-1 virus replication (Wong et al., 2011), and it’s reported CLK1 inhibition and knockdown can reduce the replication of the H1N1 influenza virus (Artarini et al., 2019). CLKs-mediated phosphorylation processes are also involved in the abnormal editing of the microtubule-associated protein tau in Alzheimer’s and Parkinson’s diseases, and CLKs inhibitors can activate the autophagic mTOR/PI3K pathway to produce therapeutic effects (Hartmann et al., 2002; Irwin, 2016; Jain et al., 2014). In addition, inhibition against CLK3 has been reported to be effective in killing Plasmodium falciparum in the infection process (Mahindra et al., 2020).

The four isoforms of CLK family (CLK1-4) are expressed in most human tissues and cells, but the expression levels of each isoform vary in different cells. For example, the expression level of CLK1 in testicular tissue is significantly lower than that of CLK2-4 (Lew et al., 1995; Nayler et al., 1997). Moreover, all CLK isoforms are usually localized in the nucleus, whereas CLK3 expressed in the testis is mostly located in the stress granules in the cytoplasm (Duncan et al., 1995). These differences in the tissue distribution of CLKs also suggest that there are needs to develop selective inhibitors for this target.

**FIGURE 1:**
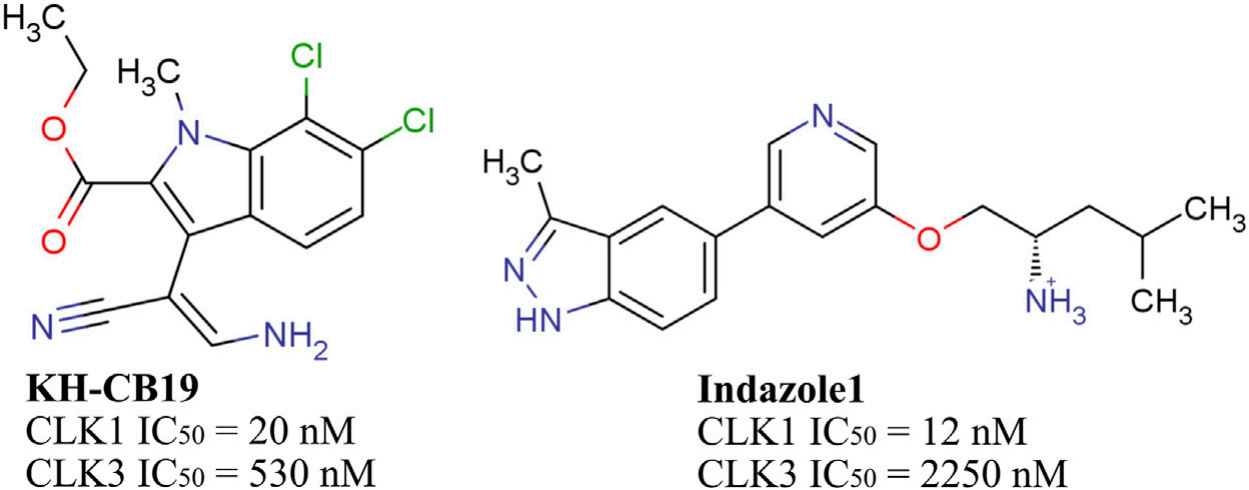
Structures and bioactivities of selective CLK1 inhibitors.

Although the four CLK isoforms differ in functions and expression levels in different tissues, they own very similar sequence and spatial structure, which is challenging for the development of selective drugs. Thus, in the case of discovering selective inhibitors towards proteins that own alike spatial structure and similar binding pocket, we hypothesize that key amino acid residues in the binding pocket are closely related to the selectivity of the inhibitor, which may affect the entry or binding of the ligand. Therefore, we expect to provide a new reference for the development of selective CLK1 inhibitors from the perspective of simulations. Two inhibitors (Indazole1, KH_CB19) with significantly different inhibitory activities against CLK1 and CLK3 were selected for investigation (Figure 1) (Moyano et al., 2020).

In this study, large-scale molecular dynamics (MD) simulations for the optimal conformation of protein-ligand complexes generated by molecular docking were performed at the atomic level in a solvent intracellular environment. The changes in the conformation of the complexes within 100 ns MD and the intensity of the interactions with key amino acid residues involving ligands were investigated to reveal the dynamical behavior of the complexes as well as the binding patterns of the ligands. Moreover, to further analyze the factors affecting the binding specificity of CLK1, binding free energy calculations, alanine scanning mutation, and quantum mechanism (QM) were applied. The computational perspective will contribute to the development of selective CLK1 inhibitors.

## Materials and methods

### Protein and ligand preparation

The crystal structures of CLK1 and CLK3 obtained from the RCSB Protein Data Bank were imported into the Schrödinger software package and processed by the Protein Preparation Wizard module (Chen et al., 2022), including removal of water molecules, construction of missing side-chain atoms, complementation of hydrogen atoms, optimization of protein conformation and relaxation of the spatial block by OPLS3 force field (Harder et al., 2016). Other chains in the crystal structure that are identical to the protein A chain were deleted.

The ligands used for molecular docking were drawn in ChemAxon Marvin Sketch (ChemAxon, 2014) and optimized by the LigPrep module (Wang and Cronan, 2004), including the generation of various conformations possible at pH 7.0 ± 2.0 and relaxation by OPLS3 force fields. Finally, each molecule generated more than two different conformations for molecular docking.

### Alignment of protein structure and sequence

The structural data of human CLK1/3 protein were acquired from RCSB Protein Data Bank (CLK1 ID: 6G33 (Heroven et al., 2018), CLK3 ID: 2WU6 (Fedorov et al., 2011)), which were imported into Discovery Studio 3.0 software package (Accelrys, San Diego, CA, United States (Chisuga et al., 2022)) for protein superposition and sequence alignment. The aligned 3D structures were used to observe differences between CLK1 and three pockets.

### Protein contacts atlas

Asteroid maps of ligand-CLK protein interactions were obtained through an online service (URL: https://www.mrc-lmb.cam.ac.uk/) to analyze key contacts and ligand binding characteristics of CLK proteins.

### Molecular docking

Molecular docking studies were performed by Glide 9.7 with XP (extra precision) mode (Du et al., 2020; Halgren et al., 2004). Glide receptor grid files were generated from the spatial locations defined by the co-crystalline ligands. The co-crystalline ligands were firstly docked to verify the grid files, and the results showed RMSD values of 0.194 Å (for PDB 6G33) and 0.441 Å (for PDB 2WU6). To evaluate the representativeness of the crystal structures used, we generated predicted structures of the same proteins using AlphaFold3 and compared them with the experimental structures (RMSD_CLK1_ = 1.324 Å, RMSD_CLK3_ = 1.593 Å). The results showed that the predicted structures were highly consistent with the experimental structures at key active sites (Supplementary Figure S3). Then the two selective CLK1 inhibitors were docked onto CLK1/3 separately, to obtain the putative structures of the four protein-ligand complexes as well as docking scores. The protein-ligand affinity of the four complexes was analyzed by calculating the Glide energy, docking score, and other parameters. The docked complexes were used for molecular dynamics simulations.

### Molecular dynamics simulations

Molecular dynamics (MD) simulations were performed by the Desmond 3.7 component of the Schrödinger package (Yi et al., 2021). First, an SPC solvent model was used around the protein-ligand complexes and an appropriate amount of Na + counter ions were added to the solvent to neutralize the whole system, generating an orthorhombic box for the dynamics simulations. Then, the system was relaxed by the OPLS3 force field (Harder et al., 2016). Then the whole system was minimized by the hybrid algorithm of steep descent and limited-memory Broyden-Fletcher-Goldfarb-Shanno (LBFGS) with the maximum number of iterations set to 5,000. The default settings in Desmond were used: the integration timestep was set to 2 fs, non-bonded interaction cut-offs were 9.0 Å for both Van der Waals and electrostatic interactions, with long-range electrostatics treated using PME. The Martyna-Tobias-Klein (MTK) barostat was employed for pressure control, and the Nose-Hoover thermostat was used for temperature regulation. Finally, 100 ns MD simulations of the above-prepared cubic orthorhombic system were performed under the NPT (constant number of particles, pressure, and temperature) system. Specifically, The NPT ensemble with the temperature 300 K and a pressure 1 bar was applied in all runs. The production MD simulation was then carried out for 100 ns with a time interval of 4.8 ps between frames. Furthermore, to better mimic physiological conditions, the solvent system was neutralized and supplemented with Na^+^ and Cl^−^ ions to achieve a final ionic concentration of 0.15 M (Dashtbin et al., 2024; Ganjali and Fogolari, 2023; Gholami et al., 2024; Hassan Omrani et al., 2025). The potential energy (U), root mean square deviation (RMSD), root mean square fluctuation (RMSF), and protein-ligand interactions were monitored during the whole simulations. After statistical analysis for the output data, the degree of stability for each complex and the key interacting residues involved in the binding was determined (Huang et al., 2020; Wang et al., 2019).

### Analysis of MD trajectory

To analyze the trajectory data from the MD simulations, RMSD values were calculated to examine the stability of the protein-ligand complexes as a whole, and RMSF values were detected to investigate the stability of each CLK residue throughout the simulations.

RMSD values were calculated according to the following Equations 1-3:

$${\text{RMSD}}_{\left(\mathrm{Pi},\mathrm{Pr}\right)}={\text{RMSD}}_{\left(\mathrm{Pi},\mathrm{Po}\right)}-{\text{RMSD}}_{\left(\mathrm{Pn},\mathrm{Po}\right)}+{\text{RMSD}}_{\left(\mathrm{Pn},\mathrm{Pr}\right)}$$ (1)

$${\mathrm{RMSD}}_{X}=\sqrt{\;\frac{1}{T}\;\sum_{j=1}^{N}\;{\left[p_{j}^{\prime }\left(t_{i}\right)\;–\;p_{j}\left(t_{\mathrm{ref}}\right)\right]}^{2}}$$ (2)

RMSF values were calculated according to the following formulas:

$${\mathrm{RMSF}}_{i}=\sqrt{\;\frac{1}{T}\;\sum_{t=1}^{T}\left\{{\;\left[p_{j}^{\prime }\left(t_{i}\right)\;–\;p_{j}\left(t_{\mathrm{ref}}\right)\right]}^{2}\right\}}$$ (3)

In the above formulas, the function p_j_' (t_i_) represents the position parameters of the complex atoms at the time i, and the function p_j_ (t_ref_) represents the position parameters of the complex atoms at the reference time, where the first frame of the simulations is used for the reference time. In Formula 1, P_i_ denotes the pose of the complex at time i, P_n_ is the pose of the terminal complex, P_o_ is the pose of the initial complex, and P_r_ is the pose of the reference complex. In Formula 2, N represents the number of atoms to be calculated. In Formula 3, T is the total duration of the calculation, which is 100 ns; the curly brackets indicate the average of the squared distances taken in the selection of the atoms in the residues (Huang et al., 2020; Wang et al., 2019).

To assess the importance of the hydrogen-bonding interactions involved in the MD simulations for several key residues, the distances of these hydrogen bonds were also measured.

### Dynamic cross-correlation matrix (DCCM)

In order to analyze the correlation between the motion of individual residues in the whole MD simulation with other residues, Desmond trajectory was used to calculate and generate the DCCM (vander Spoel et al., 2010; McCammon, 1984). To reduce statistical noise and to avoid the statistical impact of side-chain differences of different amino acid residues, we considered only the α-carbon atoms of the residue backbone for the calculation.

The interrelationships between CLK residues were described by covariance matrices, and the covariance matrix element C_ij_ was calculated by the following Equation 4:

$$C_{\mathrm{ij}}=<{\Delta r}_{i}*{\Delta r}_{j}>/\sqrt{<{\Delta r}_{i}^{2}>{*<\Delta r}_{i}^{2}>}$$ (4)

In the above equations, Δr_i_ and Δr_j_ denote the displacements of the average positions of atoms i and j with respect to time, respectively, and the pointed brackets denote the average value of the MD simulation process. the values of Cij range from −1.0 for perfectly positive correlation to +1.0 for perfect correlation.

### MM-GBSA calculation

Molecular mechanics generalized Born model and solvent accessibility (MM-GBSA) is an important method for calculating the binding free energy of ligands towards proteins and assessing the accuracy of molecular docking, which was calculated by using Prime component of Schrödinger package including the coulomb binding energy, covalent binding energy, hydrogen-bonding energy (H-bond), lipophilic energy (Lipo), pi-pi packing energy (Packing), generalized born electrostatic solventization energy (Solv GB), van der Waals energy (vdW), etc (Chisuga et al., 2023). 1,000 frames of conformations extracted from the simulation trajectory were used in the MM-GBSA calculation to evaluate the binding free energy performance throughout the MD simulation. In addition, the conformations were extracted from the 100 ns MD simulations for average binding free energy calculation according to the following Equations 5-7:

$${\Delta G}_{\mathrm{bind}}={\Delta G}_{\mathrm{complex}}-\left({\Delta G}_{\mathrm{protein}}+{\Delta G}_{\mathrm{ligand}}\right)$$ (5)

$${\Delta G}_{\mathrm{bind}}=\Delta H-\left({\Delta G}_{\mathrm{solvation}}+T\Delta S\right)$$ (6)

$$G_{\mathrm{bind}}={\Delta E}_{\mathrm{MM}}+{\Delta G}_{\mathrm{GB}}+{\Delta G}_{\mathrm{SA}}-T\Delta S$$ (7)

ΔS in the formula is the entropy change during the sampling of the ligand-binding conformation, ΔH is the enthalpy change (Huang et al., 2020; Wang et al., 2019).

### Alanine scanning mutagenesis

Alanine scanning mutagenesis (ASM) is a method for detecting the importance of specific amino acid residues in protein-ligand interactions by point mutation, which has been reported as an efficient alternative to physical experiments (Ramadoss et al., 2016). The study was conducted by mutating the amino acid to alanine, which is the shortest while retaining α-carbon chirality. Mutation to alanine does not change the conformation of the protein backbone and maximizes the removal of the side chain for the residue, thereby examining its contribution to the binding free energy. The operation was performed to calculate the change in free energy of binding (ΔΔG_bind_) when a wild-type amino acid residue is mutated to alanine. A higher positive value indicates a significant weakening effect on ligand binding, which highlights the importance of the mutated residue in protein-ligand binding (Wang et al., 2019).

The change of binding free energy was calculated by the following Equation 8:

$${\Delta \Delta G}_{\mathrm{bind}}={\Delta G}_{\mathrm{bind},\mathrm{mutant}}-{\Delta G}_{\mathrm{bind},\mathrm{wild}\;\mathrm{type}}$$ (8)

### Pharmacophore modeling

To analyze the differences in the pharmacophore characteristics of CLK1/3, the mean conformation of MD simulations were extracted to generate the pharmacophore model using LigandScout 4.3 software (Alamri and Alamri, 2019; Kainrad et al., 2019), which was mainly based on hydrogen bonding, electrostatic, van der Waals, hydrophobic, and other ligand-protein interactions to generate pharmacophore.

### Hirshfeld surface analysis

To investigate the compositions of the protein-ligand complex interaction, Hirshfeld surface analysis reveals the strength of the individual interactions by calculating the electron density distribution of the atomic fragments at the contact surface of the ligand and the protein (Spackman and Jayatilaka, 2009). The method is able to clearly show the non-covalent interactions within the binding pocket, thus facilitating the analysis of the protein-ligand interactions involving residues. In this study, the conformations of the last frame of the MD simulations were extracted. The calculations were performed using Multiwfn V3.9 (Lu and Chen, 2012) and the results were visualized by VMD software (Humphrey et al., 1996).

## Results and discussion

### Comparison of CLK1 and CLK3 structures

**FIGURE 2:**
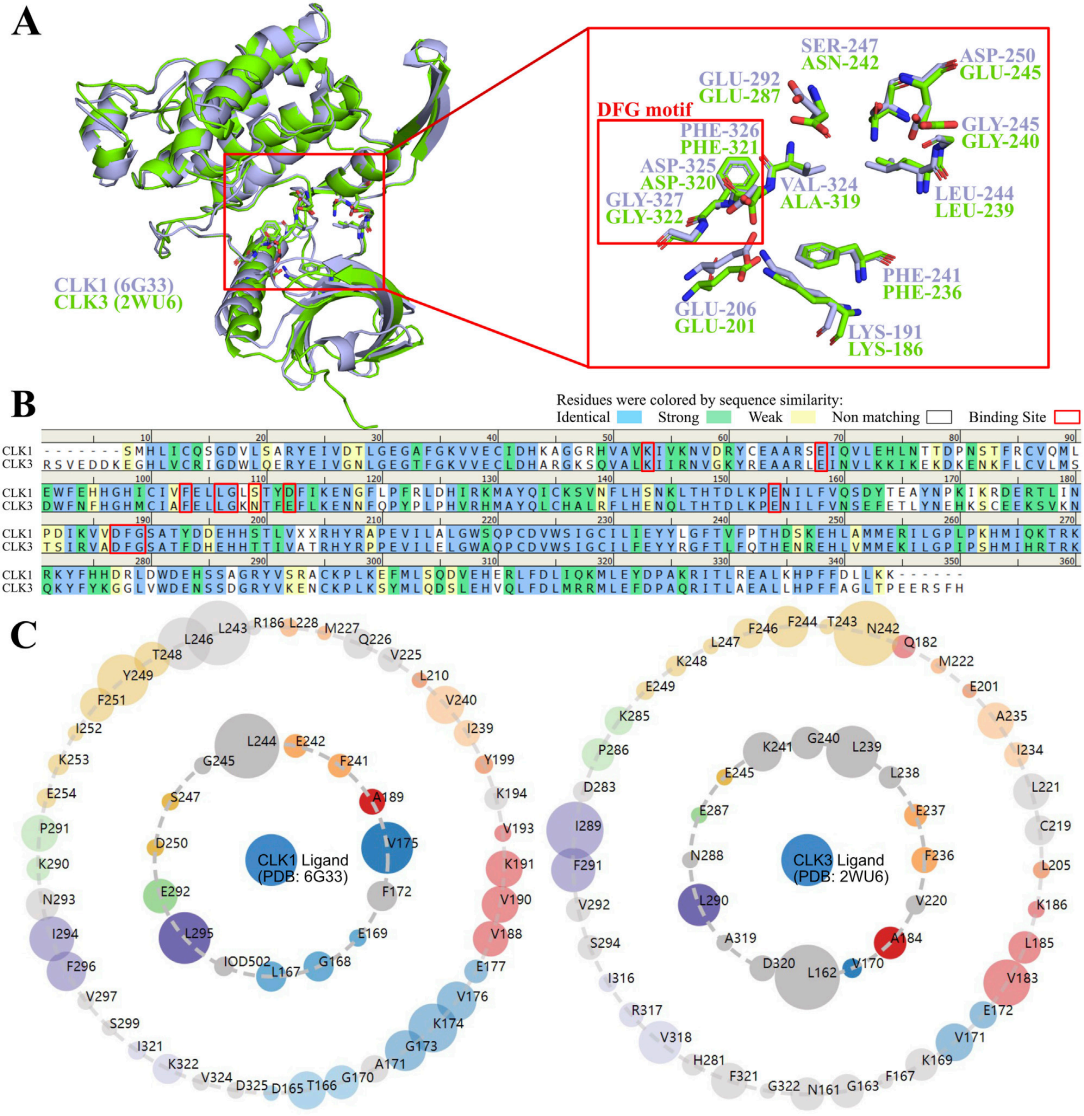
Comparison of sequences and structures of CLK1 and CLK3. **(A)** Protein structure alignment of human CLK1 (PDB ID: 6G33, purple ribbon) and CLK3 (PDB ID: 2WU6, green ribbon). **(B)** Sequence alignment between CLK1 and CLK3. Characters with blue backgrounds indicate identical residues, green for strongly similar residues, yellow for residues with weak similarity, and white for non-matching residues. Key amino acid residues involved in ligand binding are shown in red boxes. **(C)** Asteroid plots formed by Protein Contact Atlas. The inner shell residues are immediate residues that form intermolecular contacts with CLK ligands. The outer shell residues indirectly contact CLK ligands. The size of the nodes in the inner and outer concentric circles denotes the number of atomic contacts, and the residues are colored according to secondary structures.

CLKs own similar typical conserved structures as other serine/threonine protein kinases, with the ATP-binding site within the gap between N- and C-lobes with highly similar tertiary structures (Figure 2A), which are connected by a hinge region. Among CLK family members, CLK1 and CLK3 are very close to each other in the kinase evolutionary tree (Supplementary Figure S1). The results of sequence alignment showed 59.0% sequence identity and 77.3% sequence similarity between CLK1 and CLK3, and both CLK isoforms own conserved amino acid residues within their binding pockets (Figure 2B), which are also clearly reflected by the asteroid map (Figure 2C), and the highly similar binding pocket residues bring a challenge for the development of selective CLK1 inhibitors. The Asp-Phe-Gly (DFG) motif locates in the hinge region of CLK1 and CLK3 pocket that is common in kinase structure, and the ATP-competitive CLK1 inhibitors like Indazolel1 and KH_CB19 interact with the DFG-in state that ATP is allowed in. It is noteworthy the residues preceding DFG motif in CLK protein sequence, denoted by DFG-1, consists of different VAL324 (CLK1) or ALA319 (CLK3), which causes unequable hydrophobic environments between CLK1 and CLK3 pockets. In addition, potential electrostatic interactions from ASP250 (CLK1) may contribute to the selectivity of CKL1 inhibitors.

### Binding affinities and patterns of selective CLK1 inhibitors

The binding patterns and affinities of compounds two inhibitors towards CLK1 and CLK3, respectively, were predicted using molecular docking and were valuated utilizing scoring functions such as XP GScore and Glide emodel.

Indazolel1 possessed better docking score towards CLK1 (−15.059 kcal/mol) than KH_CB19 (−8.312 kcal/mol), confirming Indazolel1 exhibited better binding affinity against CLK1 over CLK3 (Table 1).

**TABLE 1:** Biological activities (IC_50_) and Glide docking scores of CLK1 and CLK3 inhibitors.

Inhibitor	CLK 1		CLK 3	
	IC_50_ (nM)	Docking score (kcal/mol)	IC_50_ (nM)	Docking score (kcal/mol)
Indazolel1	12	−15.059	2250	−5.486
KH_CB19	20	−8.312	530	−6.850

**FIGURE 3:**
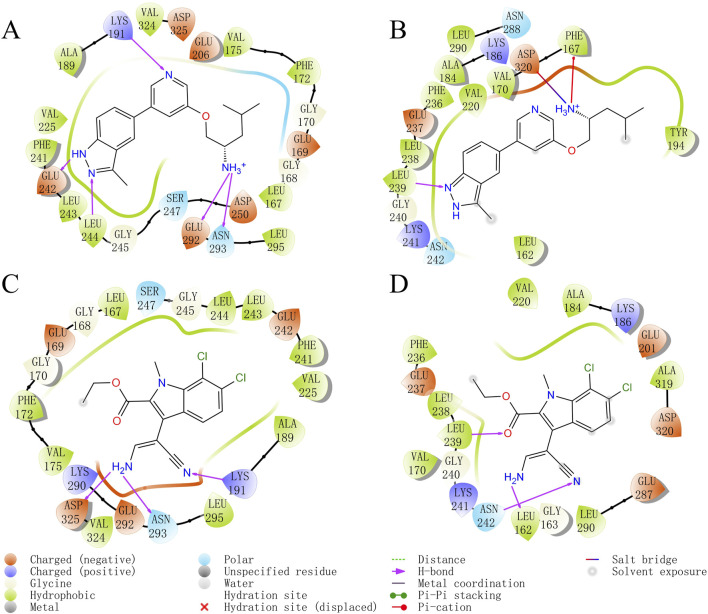
Predicted binding patterns of CLK1/3 inhibitors. **(A)** CLK1/Indazolel1, **(B)** CLK3/Indazolel1, **(C)** CLK1/KH_CB19, and **(D)** CLK3/KH_CB19.

As shown in the visualized residue contact patterns generated by Glide docking (Figure 3), Indazolel1 and KH_CB19 generated conserved hydrogen-bonding interactions with CLK1 residues such as ASN293 and LYS191, but only formed a conservative hydrogen bond with LEU239 of CLK3. In addition, the hydrophobic domain inside CLK1 pocket (surrounded by residues VAL225 to LEU244) is capable of interacting with the hydrophobic moiety of Indazolel1 and KH_CB19.

**FIGURE 4:**
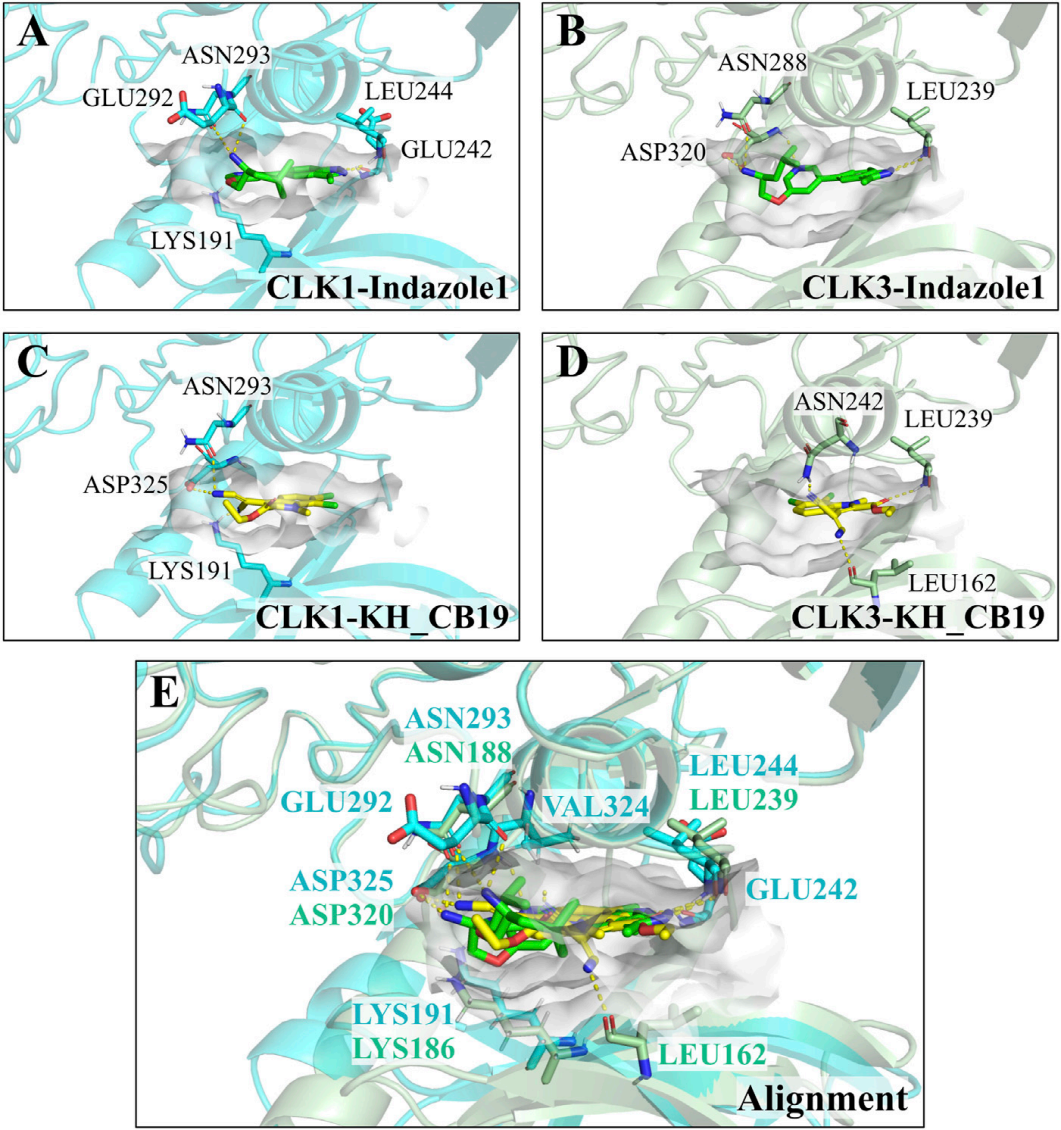
Snapshot of docked poses. **(A, B)** Indazolel1 (green stick) within CLK1 (blue ribbon) and CLK3 (green ribbon) pockets. The binding pockets are represented as gray surface. **(C, D)** KH_CB19 (yellow stick) within CLK1 and CLK3 pockets. **(E)** Poses alignment.

Comparing to the hydrogen bond interactions between the key residue LYS191 of CLK1 and both compounds, the corresponding residue LYS186 of CLK3 fails to form the similar H-bond interaction with the compounds, due to the formidable faraway distance between compounds and LYS186 of CLK3 (Figure 4), thereby it’s hypothesized that the stable hydrogen bonding interactions with LYS191 of CLK1 may be crucial in differentiating the inhibitory selectivity towards CLK1 over CLK3, and molecules with longer electronegative groups may establish new hydrogen-bonding interactions with LYS186 of CLK3 which may decrease the inhibitory selectivity between CLK1 and CLK3. Furthermore, within the hydrophobic region of CLK1 pocket, the benzo pyrazolyl of Indazole1 is able to form additional stable H-bonds with GLU242 and LEU244 of CLK1 (Figure 4A), which may be beneficial for the prior inhibition towards CLK1.

In the hydrophobic area of CLK1 pocket, the key residue VAL324 is located above the CLK1 binding pocket (Figure 4E) which formed a stable hydrophobic interaction with the benzo pyrazolyl group in Indazole1 and the benzimidazolyl group in KH_CB19, whereas CLK3 lacks such hydrophobic interaction due to the valine be substituted by an alanine. Varying this position leads to a decrease in hydrophobicity within the binding pocket, which may alter the ligand-protein interaction pattern and should be considered in the development of selective inhibitors.

In summary, as with remarkable inhibition and optimal docking performance towards CLK1, Indazole1 formed stable interactions with LYS191 and VAL324 of CLK1, and additional strong contacts with several residues of CLK1 such as PHE241, GLU242, GLU292, and ASP325, contributing to the promising inhibition and selectivity, which suggests that selective CLK1 inhibitors require the presence of a hydrophobic group that interacts stably with VAL324 and an electronegative group that is just accessible to LYS191 within CLK1 pocket to generate a stable hydrogen bond.

### Molecular dynamics trajectories

**FIGURE 5:**
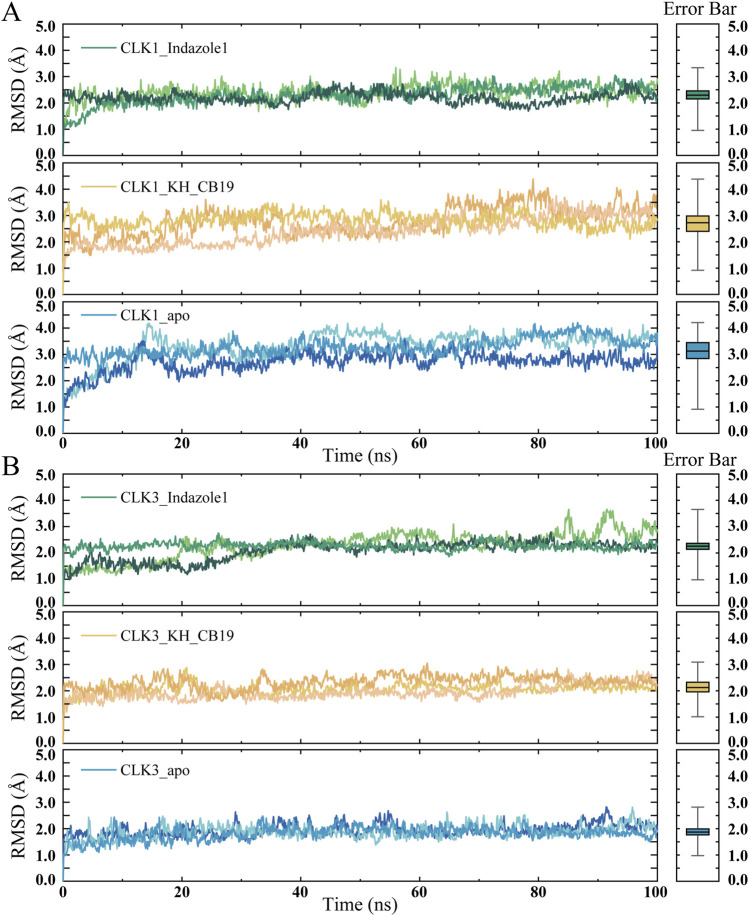
RMSD values of MD simulations. **(A)** RMSD chart of CLK1. **(B)** RMSD chart of CLK3. The three sets of repeated simulations are represented by lines of similar colors. The box-plot graph on the right side indicates the average value within 100 ns of simulations. The box extends from the 25th to 75th percentiles. The line in the middle of the box is plotted at the median. Error bar indicated the min-max value of RMSD during the simulations.

The four docked complexes and the two apo protein of CLK proteins were subjected to 100 ns molecular dynamics simulations with RMSD and RMSF values monitored. RMSD values assess the effect of ligand constraint on the protein conformation by comparing the overall stability of the complexes by quantifying the displacement values of all atoms. All the investigated systems reach a steady state at the end of the MD simulation during the 100 ns simulations. The conformations of CLK1 complexes and apo structure stabilized after 20 ns, and the significantly lower mean RMSD values of CLK1 complexes than the apo CLK1 structure suggests that both inhibitors can tightly bind with CKL1 to stabilize CLK1 conformation (Figure 5A; Supplementary Figure S4). In the RMSD curves of CLK3 complexes, KH_CB19 showed the most stable curve with RMSD value of 2.0 Å during the whole simulation. After stabilizing for up to 50 ns, the RMSD value of the CLK3 apo structure underwent a conformational change at 70 ns with increased RMSD value, and finally stabilized at 3.5 Å at the end of the simulation (Figure 5B). Moreover, the trend of the mean RMSD values is consistent with the reported bioactivity data of Indazole1 and KH_CB19 and the average RMSD value was smaller for proteins that bound small molecules compared to apo proteins suggesting that the selective CLK1 inhibitors are able to produce more stable constraint on the CLK1 conformation during the MD simulations.

**FIGURE 6:**
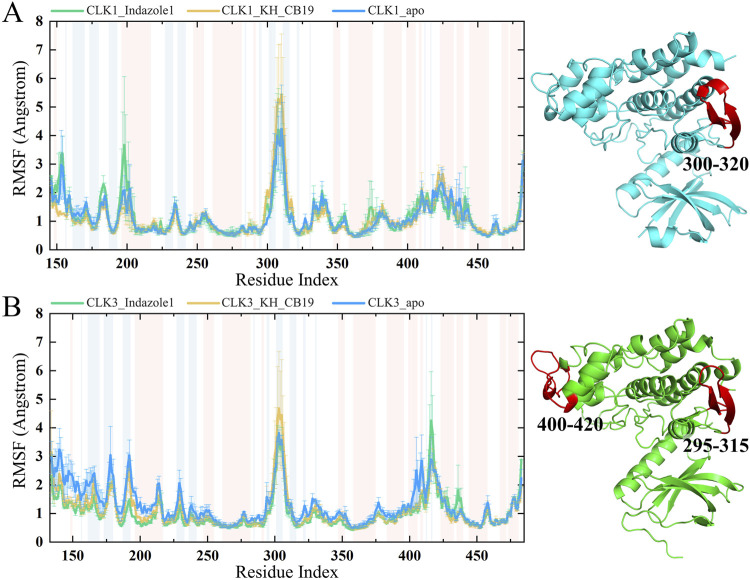
RMSF values of MD simulations. **(A)** RMSF chart of CLK1. **(B)** RMSF chart of CLK3. Error bar indicated the min-max value in the three sets of repeated simulations. Backgrounds in blue indicated residues in the β-sheet secondary structure and red ones indicated residues in the α-helix secondary structure. The red part in the three-dimensional structure of the protein ribbon on the right is the peak site of the RMSF curve.

RMSF values were monitored during the simulations to assess the stability of each residue of the backbone in the CLK complexes in 100 ns MD simulations. Except for the exposed loop region, most residues within CLK sites are more stable than the apo structure (Figure 6), such as residues VAL324 and ASP325 of CLK1, disclosing that the ligand-binding within the pocket restricted the local conformational changes of the protein. Some loop regions of the RMSF curves have large conformational changes, such as 300–320 for CLK1 and 295–315, 400–425 for CLK3, are protruding, expose to the solvent, consist or connect of random coils, lack the interaction constraints between the secondary structures within the protein.

**FIGURE 7:**
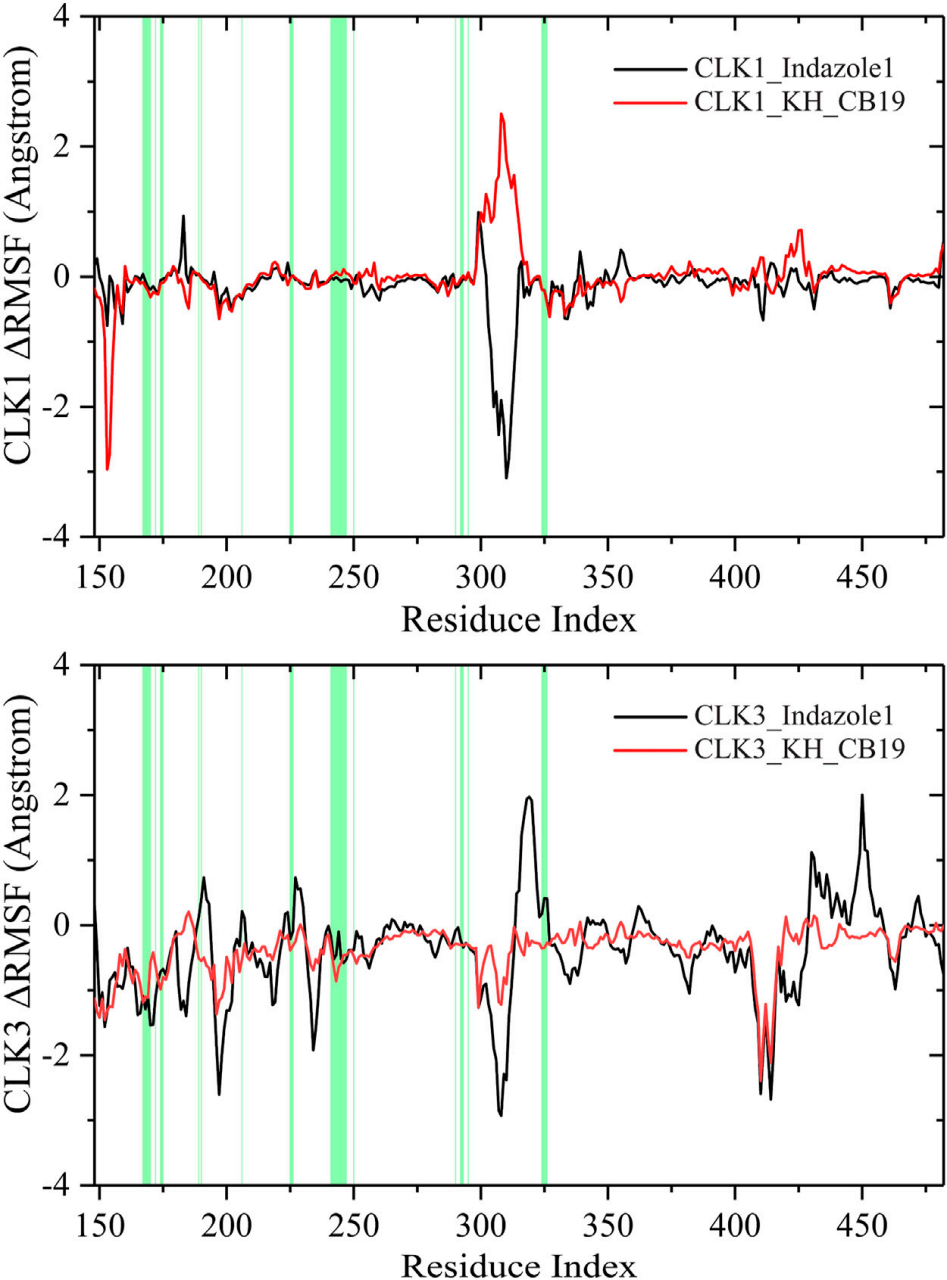
Change of RMSFs of CLK1/3 induced by Indazole1 and KH_CB19. The residues that constitute the binding pocket are highlighted by the green background. The residue index was based on CLK1 and was aligned.

The changes of RMSF values of CLK proteins (ΔRMSF) caused by the combination of Indazole1 and KH_CB19 were visualized by subtracting the value of apo from the value of the complex. As shown in Figure 7, a negative ΔRMSF value indicates that the residue conformation is stabilized by bound inhibitors compared to the apo structure. Both Indazole1 and KH_CB19 complexes display negative RMSF values for the residues located in the binding pocket, implying that both inhibitors are able to form interactions with CLK1 pocket and thus to stabilize the local residues. In addition, corresponding to the previous RMSF curves, ΔRMSF also shows substantial fluctuations at loop regions or solvent-exposed regions (domain 300–320 and 400–425) that lack secondary structure interaction constraints, and conformational changes are more random and tend to produce larger conformational changes, which manifest as peaks in RMSF values. Distinctly, the Indazole1-CLK1 complex shows negative ΔRMSF peaks in both regions, implying that Indazole1 may form a more effective conformational constraint on the loop region of CLK1 compared to KH_CB19. In conclusion, the above results show that Indazole1 tends to be better conformationally bound to CLK1 than KH_CB19, implying a better inhibitory activity against CLK1.

**FIGURE 8:**
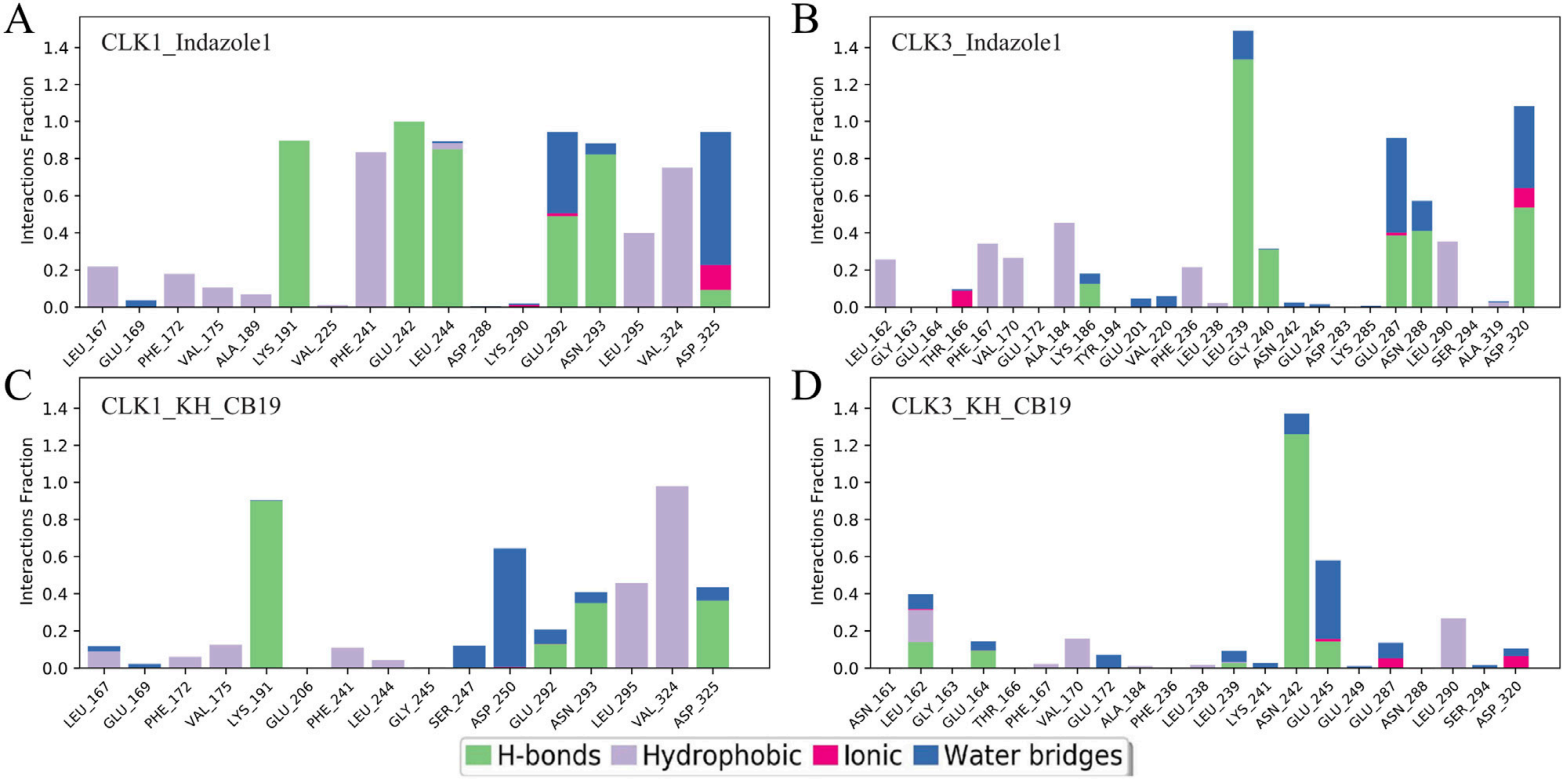
Interaction fractions of protein-ligand obtained through the MD trajectory for complexes. **(A)** CLK1/Indazolel1, **(B)** CLK3/Indazolel1, **(C)** CLK1/KH_CB19, and **(D)** CLK3/KH_CB19.

The bar charts of protein-ligand contacts also showed more strong interactions between CLK1 and both inhibitors, with significantly higher intensity and interaction fractions than CLK3 (Figure 8). Specifically, VAL324 of CLK1, the key selective residue DFG-1, created substantial hydrophobic interaction with the benzo pyrazolyl in Indazole1 and the benzimidazolyl in KH_CB19 deep in the binding pocket, while CLK3 lacked this strong hydrophobic effect due to the change of valine to alanine resulting in a substantial decrease in the ligand affinity of the CLK3 binding pocket, which is consistent with the known experimental data and may be an important selective factor. Furthermore, LYS191 of CLK1 generated strong hydrogen bonds with both ligands, while the corresponding LYS186 of CLK3 had almost no such interaction due to a conformational difference in the hinge region of the protein: the C-terminus of the β4 sheet, where the lysine is located, is overall far from the ligand-binding site, making it less likely for the inhibitor to generate an effective hydrogen binding contact with CLK3-LYS186. In conclusion, the two CLK1-exclusive and stable protein-ligand interactions described above are essential for the selectivity of Indazolel1 and KH_CB19.

To further assess the role of key residues, three hydrogen bonds that play an important role in the interactions of CLK1 complexes were monitored and the distances were calculated at which the compounds were in contact with the residues. The CLK3-KH_CB19 complex is not involved in the interactions of the key hydrogen-bonded amino acid residues (Supplementary Figure S2).

**FIGURE 9:**
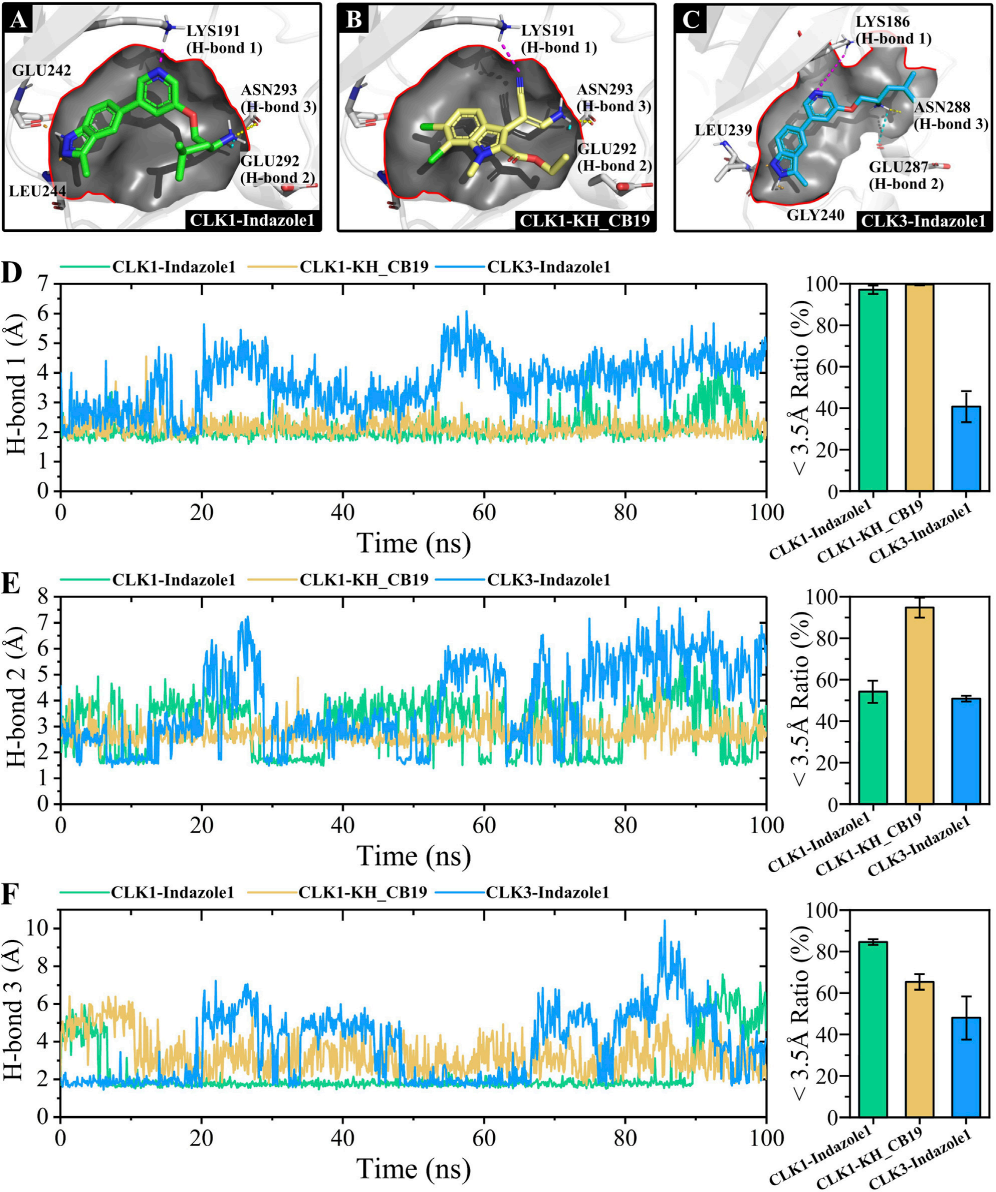
Hydrogen bonding formation and distances of CLK complexes. **(A)** Indazolel1 (green stick) binds within CLK1 pocket, **(B)** KH_CB19 (yellow stick) binds within CLK1 pocket, and **(C)** Indazolel1 (blue stick) binds within CLK3 pocket. **(D)** H-bond distances with lysine residues (purple dashed lines), **(E)** H-bond distances with the glutamate residues (blue dashed lines), and **(F)** H-bond distances with the asparagine residues (yellow dashed lines). The hydrogen-bonding interactions with different residues are indicated by dashed lines of different colors, respectively. The bar chart on the right represents the percentage of H-bond distances within <3.5Å during the simulations and the error bar exhibited the max-min value of distances in the three sets of repeated simulations.

For the key lysine residues located above the pocket, LYS191 of CLK1, Indazolel1 and KH_CB19 are all involved in hydrogen bonding interactions at an average distance of about 2 Å (Figure 9D). Although the hydrogen bonding distances of both CLK1 complexes fluctuate up to 4 Å, the CLK3-Indazolel1 conforms visibly to a much larger contact distance with the LYS186 residue than the former two, even up to 6 Å, which is beyond the effective distance for hydrogen bond formation. For the glutamine residue on the right side of the pocket, the CLK1-KH_CB19 complex formed the most stable hydrogen bond with a distance of 3 Å (Figure 9E). While the hydrogen bond formed by the CLK1-Indazolel1 complex fluctuated within 2–4 Å for most of the time, it also maintained an effective contact with ASN293. The CLK3-Indazolel1 complex also formed an effective hydrogen bond at a distance around 3 Å for the first 20 ns and 30–54 ns of the MD simulation, but it broke after 70 ns (Figure 9F). For the asparagine residue on the right, the CLK1-Indazolel1 complex maintains a more effective hydrogen bond contact of 2 Å for 10–90 ns, but it reaches a farther distance of 6 Å in the last 10 ns of the MD simulation, which is caused by a conformational change of the complex at the end of the simulation. The CLK1-KH_CB19 complex maintains a stable contact of 3 Å overall. In contrast, the CLK3 complex still shows highly unstable distance fluctuations towards ASN288.

In summary, among the hydrogen bonds involving the three key amino acids mentioned above within the binding pocket, both Indazolel1 and KH_CB19 binding CLK1 generated complexes showed more stable interactions in the effective contact range. However, the key hydrogen bonding distances of the CLK3-Indazolel1 complex often exhibit a large instability, which prevents the compound from binding efficiently to CLK3.

To further evaluate the above critical hydrogen bonding interactions, the frequency distribution of the hydrogen bonding distances of the three complexes was calculated and visualized (Supplementary Figure S5). It is obvious that the key hydrogen bonds of the CLK1-Indazolel1 complexes all act at distances near 2 Å, indicating that Indazolel1 formed strong and stable hydrogen bonding interactions with the residues. And the key interaction distances of the CLK1-KH_CB19 complex are mostly distributed within 3 Å, which also lies within the effective contact range. However, the CLK3-Indazolel1 complex has a wider distribution of distances, with only about one-third lying within 3 Å, while about half of the distances are beyond 4 Å, suggesting that Indazolel1 generates fewer effective hydrogen bonding contacts within the pocket. In conclusion, Indazolel1 was able to form effective hydrogen bonds with key amino acids of CLK1, showing a stronger affinity for CLK1. Also, the difference in the distribution of hydrogen bond distances of Indazolel1 to CLK1 and CLK3 reveals its selective inhibitory activity. The results of tracking hydrogen bond distances during MD simulations support the instability of the conformation of the inhibitor Indazolel1 within the CLK3 binding pocket, which may be caused by the difference in the hydrophobic pocket due to the discrepancy between VAL324 of CLK1 and ALA319 of CLK3.

The contacts of Indazolel1 and KH_CB19 with each residue within the CLK1 binding pocket all clearly showed a more stable and intensive contact than CLK3 in the MD simulations (Supplementary Figure S6). In addition, MD simulations were also calculated the variation properties including ligand RMSD, the radius of gyration, intramolecular hydrogen bonds, molecular surface area, solvent accessible surface area, and polar surface area, etc. (Supplementary Figure S7).

In conclusion, the results of MD simulations showed that Indazolel1 and KH_CB19 selectively higher affinity for CLK1 than CLK3, which was also reflected in the stability of the CLK1-Indazolel1 and KH_CB19 complexes. Furthermore, the stable interaction involved in LYS191 and VAL324 residues of CLK1 is also suggested to be closely related to the CLK1 and three selective mechanisms.

### Dynamic cross-correlation matix (DCCM)

**FIGURE 10:**
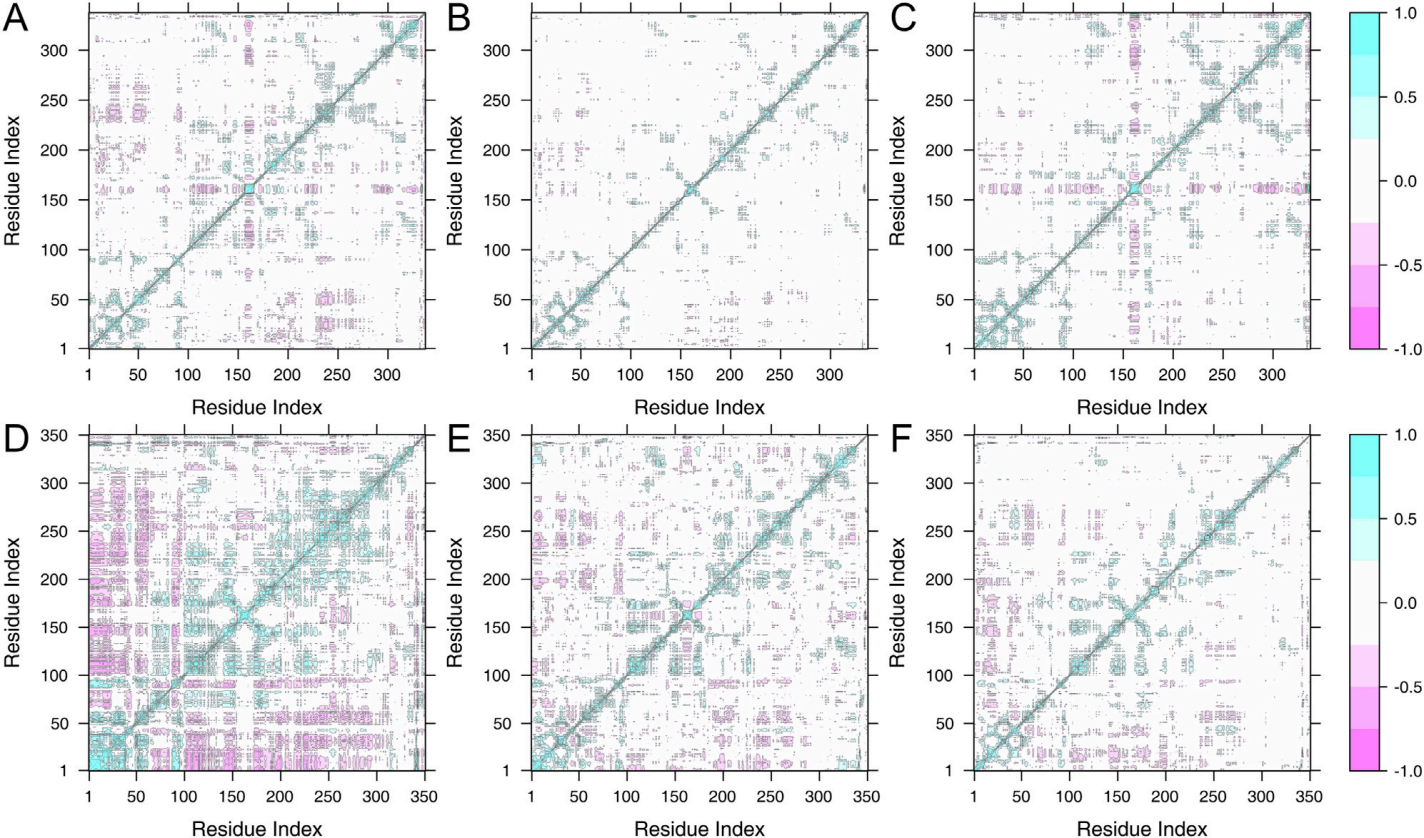
Dynamic cross-correlation matrix (DCCM) map. **(A)** CLK1 apo, **(B)** CLK1/Indazolel1, **(C)** CLK1/KH_CB19, **(D)** CLK3 apo, **(E)** CLK3/Indazolel1, and **(F)** CLK3/KH_CB19. The degree of correlation and anti-correlation is indicated by color. Cyan indicates complete correlation and purple indicates complete non-correlation.

To observe the effect of inhibitor binding on the kinetics of each residue between CLK1 and 3, the coordinates of the α-carbon atoms of the main chain of each residue in the MD simulations were used to plot the DCCM (Figure 10). Overall, the inhibitor-bound complexes in the DCCM all exhibited a decrease in both correlated and non-correlated movement due to the binding of both inhibitors resulted in an effective restraint of the protein backbone motion.

Compared to the apo structure of CLK1 (Figure 10A), a clear cross-shaped non-correlated is observed in the CLK1/KH_CB19 complex (Figure 10C), with a evidently strong positive correlation square in the central region, suggesting that the change region is unbound and the multiple residues as a whole move in non-correlation with other parts which corresponds to a random coil at the 300–320 segment of the RMSF curve, indicating that the binding of KH_CB19 does not act as a restraint on the movement of the residue backbone at this site. In contrast, the binding of Indazolel1 was able to stabilize the conformation at this site. Thereby, Indazolel1 is able to provide a stable conformational environment near the binding cavity.

### Binding free energy calculation

**FIGURE 11:**
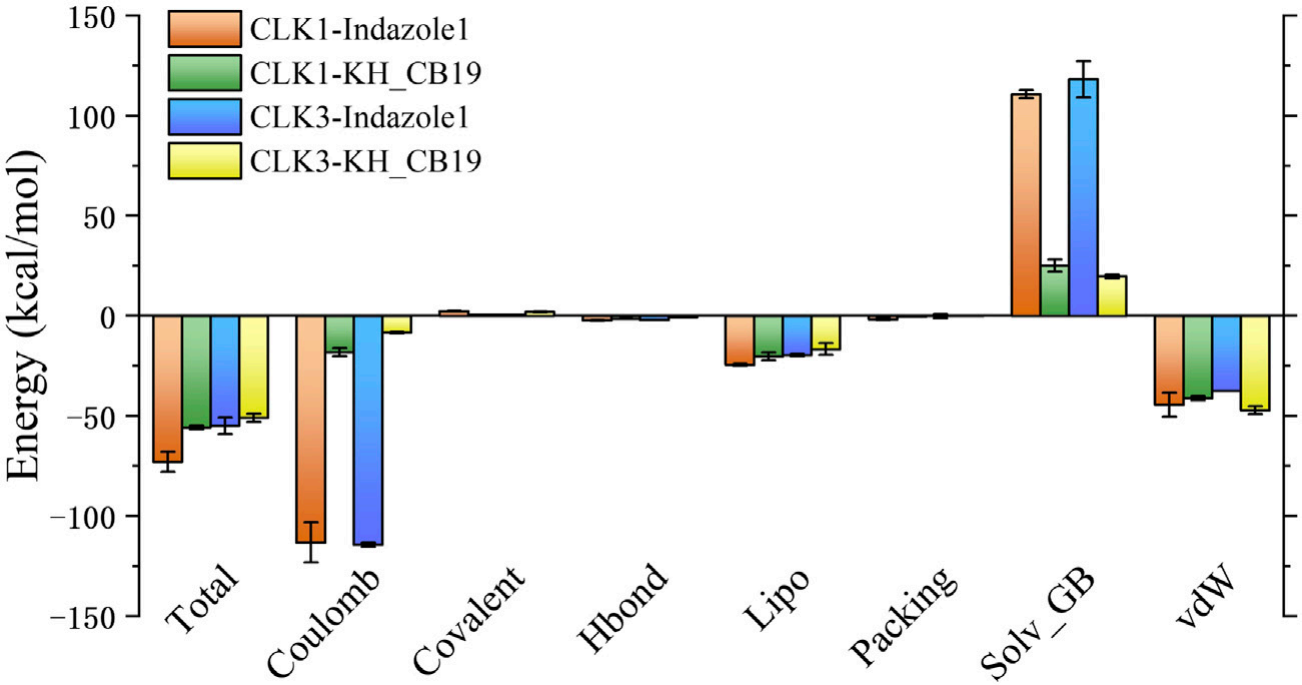
The contributions of the binding free energy between protein-ligand complexes determined by MM/GBSA calculations. Error bar indicated the min-max value in the three sets of repeated calculations.

The calculated binding free energy is consistent with Glide molecular docking, i.e., inhibitors performed better for binding to CLK1 than CLK3, reflecting the selectivity of the two inhibitors for CLK1 (Table 2; Figure 11). The binding free energy disparities between CLK1 and CLK3 complexes are mainly caused by Coulombic interactions (e.g., hydrogen bonding, salt bridges), but this is also affected by the side effects of polar solvation energy.

**TABLE 2:** Statistical analysis of MM/GBSA results.

Energy	CLK1 (kcal/mol)		CLK3 (kcal/mol)	
	Indazole1	KH_CB19	Indazole1	KH_CB19
Total	−72.95	−55.72	−54.91	−50.95
Coulomb	−113.24	−18.12	−114.30	−8.34
Covalent	2.27	0.53	0.48	1.98
Hbond	−2.17	−1.56	−2.01	−0.61
Lipo	−24.49	−20.21	−19.67	−16.58
Packing	−1.70	−0.33	−0.11	−0.01
Solv_GB	110.77	25.12	118.25	19.72
vdW	−44.38	−41.14	−37.56	−47.09

**FIGURE 12:**
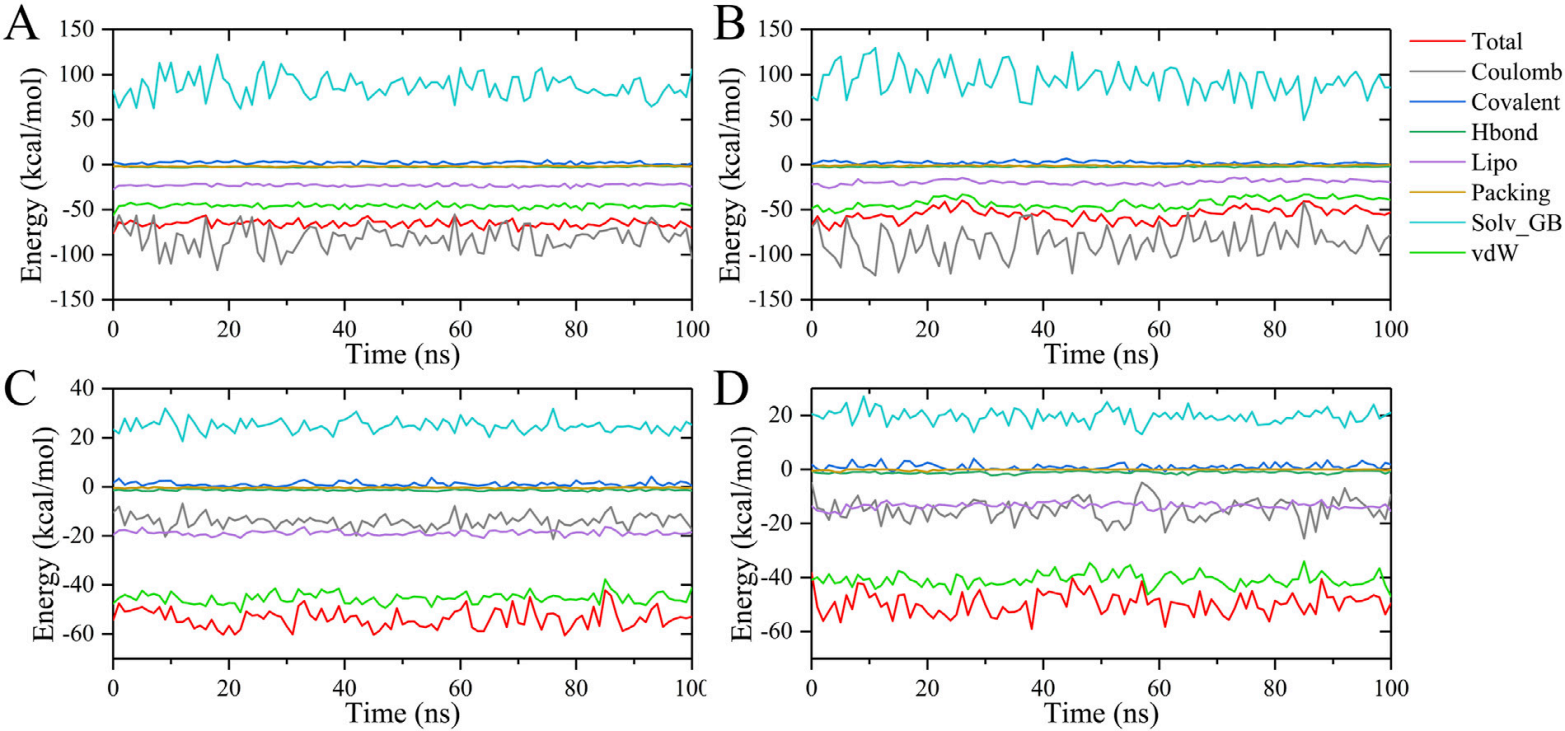
Binding free energy and its contribution in 100 ns MD simulations. **(A)** CLK1/Indazolel1, **(B)** CLK3/Indazolel1, **(C)** CLK1/KH_CB19, and **(D)** CLK3/KH_CB19.

To evaluate the changes in binding free energy in the MD simulation, 100 frames of dynamical conformations were extracted for the calculations (Figure 12). The calculated results are similar to the MD simulated RMSD curves, without steep fluctuations, reflecting the overall stability of the four complexes during the simulations. Also, the curves show the same trend as the average binding free energy, i.e., the inhibitors all bind to CLK1 at higher energies than CLK3 on average. In addition, the CLK1/3-Indazolel1 complexes generated larger Coulomb effects throughout the simulations but were partially offset by the polar solvation energy. In conclusion, the results of the combined free energy calculations are consistent with molecular docking, which further provides the accuracy of the computational perspective.

### Alanine scanning mutagenesis analysis

CLK1 amino acid residues involved in interaction with fractions greater than 0.8 in MD simulations were mutated to alanine, to examine the contribution of their side chains in the binding free energy. A positive ΔΔG value indicates a decrease in binding free energy after mutation of the amino acid residue to alanine, and a higher positive value demonstrates the importance of the interaction between the side chain of the wild-type residue and the inhibitor. A negative ΔΔG value indicates that the mutation increases the binding free energy and that the amino acid residue interacts with the ligand mainly through the main chain atoms while the side chain contributes less.

**FIGURE 13:**
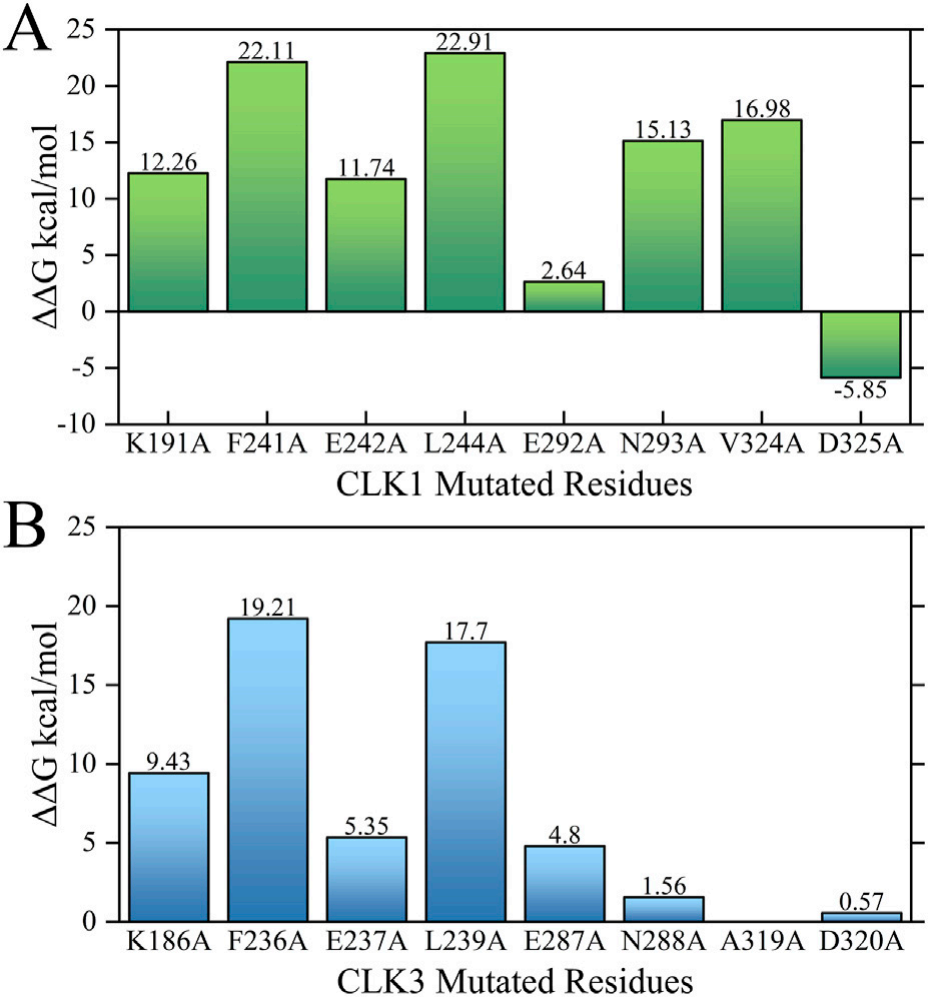
Alanine scanning mutagenesis analysis of **(A)** CLK1/Indazolel1 and **(B)** CLK3/Indazolel1 complexes. The positions of the residues in the two figures correspond to each other.

Amino acid residues K191, F241 and L244 of CLK1, and K186, F236 and L239 of CLK3 all possessed high positive ΔΔG values, indicating the dominance of their side chains in the generation of hydrogen bonds or hydrophobic interactions (Figure 13). Although these three residues contribute differently to hydrogen bonding or hydrophobic interactions in CLK1 and CLK3, mutation of their side chains would have a large change in the binding conformation due to the close relationship between their side chains and the spatial composition of the ligand-binding pocket. Therefore, the mutation results for all the three residues above show a large reduction in binding free energy. The positive ΔΔG values of CLK1-E242, N293 residues are higher than corresponding CLK3 residues E237, N288, indicating that the side chains of the two residues are more important in the hydrogen bonding interaction of CLK1 with the ligand. The lower positive ΔΔG values of CLK1-E292 and CLK3-E287 indicate that the formation of hydrogen bonds and water-bridge interactions are mainly related to the main chain, while the contribution of the side chains is smaller. The high positive value of ΔΔG for CLK1-V324 indicates that the key hydrophobic role here relies mainly on the contribution of the isopropyl side chain of valine. Interestingly, the wild-type CLK3-A319 is originally alanine, and the mutation results again demonstrate the importance of the hydrophobic role here. The side chain change from isopropyl to methyl leads to the loss of hydrophobic interaction with the benzo pyrazolyl in Indazole1. Finally, CLK1-D325 showed negative values of ΔΔG in mutation stimulations, suggesting that the side chain of this residue is not involved in the formation of protein-ligand interactions and even partially hinders the generation of water-bridge interactions between the main chain and the ligand.

### Pharmacophore features for selective inhibition of CLK1/3

**FIGURE 14:**
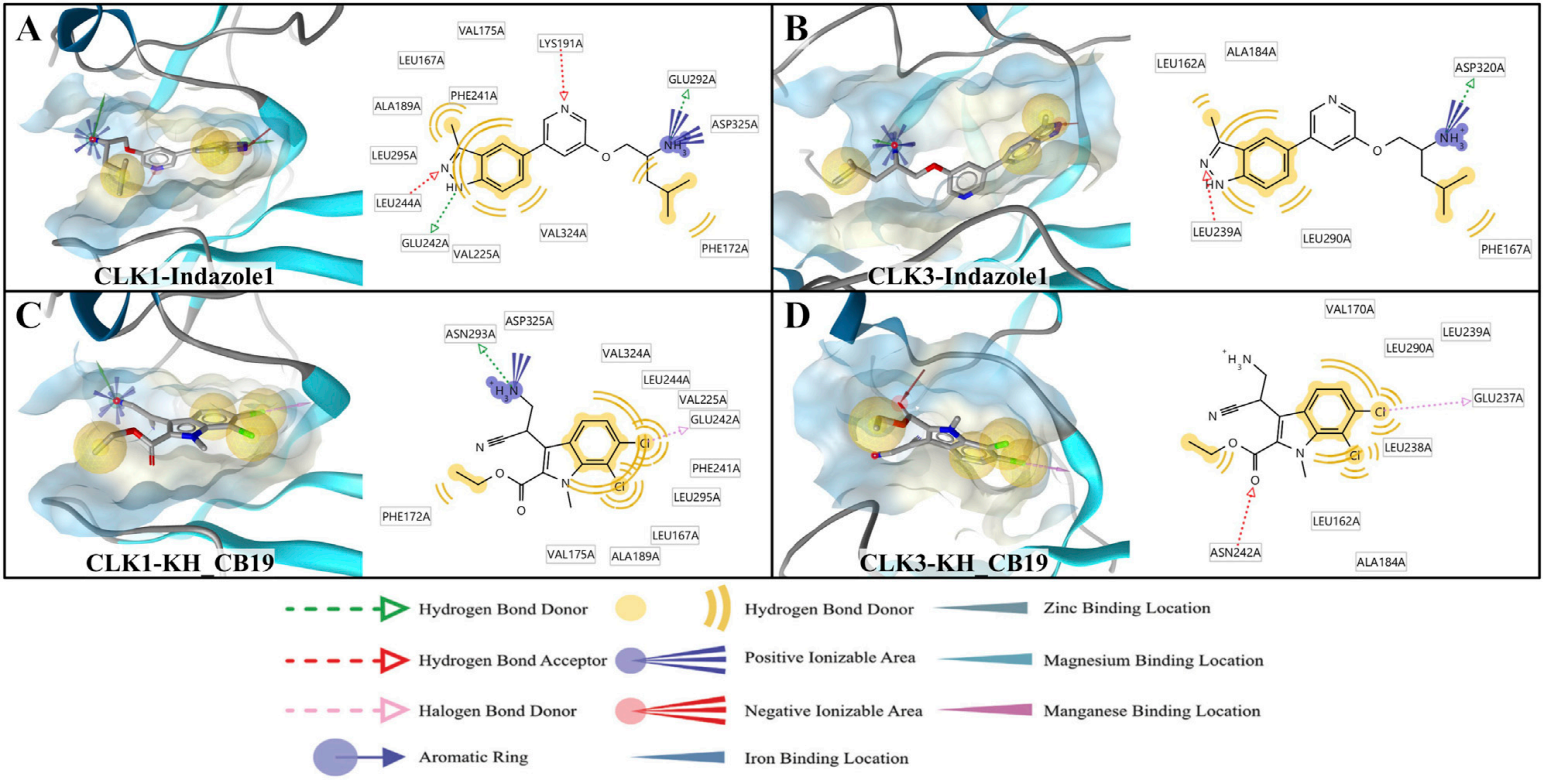
Structure-based pharmacophore models. **(A)** CLK1/Indazolel1, **(B)** CLK3/Indazolel1, **(C)** CLK1/KH_CB19, and **(D)** CLK3/KH_CB19. Hydrophobic and hydrogen bond acceptor interactions are depicted as yellow spheres and red arrows, respectively.

The characteristic pharmacophores generated by LigandScout reveal (Figure 14) CLK1 inhibitor contains a hydrophobic space consisting of two or three hydrophobic pharmacophores, mainly surrounded by PHE241, LEU295, VAL324 of CLK1. Moreover, in this hydrophobic space, the two nitrogen atoms in the benzo pyrazolyl of Indazole1 respectively generated a hydrogen bond acceptor and a hydrogen bond donor toward the CLK1-GLU242, LEU244 residues, which may be one of the important factors for the high inhibitory activity of CLK1. In addition, the pyridyl in Indazole1 generated a hydrogen-bonded acceptor to CLK1-LYS191. According to molecular docking and MD simulations, the cyano group in KH_CB19 also generated hydrogen bonds with CLK1-LYS191, where the pharmacophore was not shown. Finally, Indazole1 and KH_CB19 both have a hydrophobic pharmacophore consisting of an amino group generating a positive ionizable area interacting with GLU292 and ASN293 by a hydrogen bond donor. And the two inhibitors both have a hydrophobic pharmacophore interacting with PHE172 in the entrance of the CLK1 binding pocket.

As with the molecular docking results, CLK3-LYS186 did not generate a hydrogen bond with the nitrogen atom in the pyridyl in Indazole1 due to the distance limitation caused by the conformational difference between the two proteins. In addition, due to conformational differences between the CLK1 and three binding pockets, the pyridyl in Indazole1 is distant from CLK3-GLU237, generating a hydrogen bond only with LEU239. Also, since DFG-1 within the hydrophobic space of the CLK3 binding pocket changed valine to alanine, thereby reducing the hydrophobic effect inside the pocket than CLK1, which is an important reason for the difference in conformation of Indazole1 between the CLK1 and three binding pockets. The absence of the three important pharmacophores mentioned above leads to a substantial reduction in the inhibitory bioactivity of Indazole1 against CLK3, which is the basis for selective inhibition.

### Shape complementarity of inhibitors within CLK1 and CLK3 pockets

**FIGURE 15:**
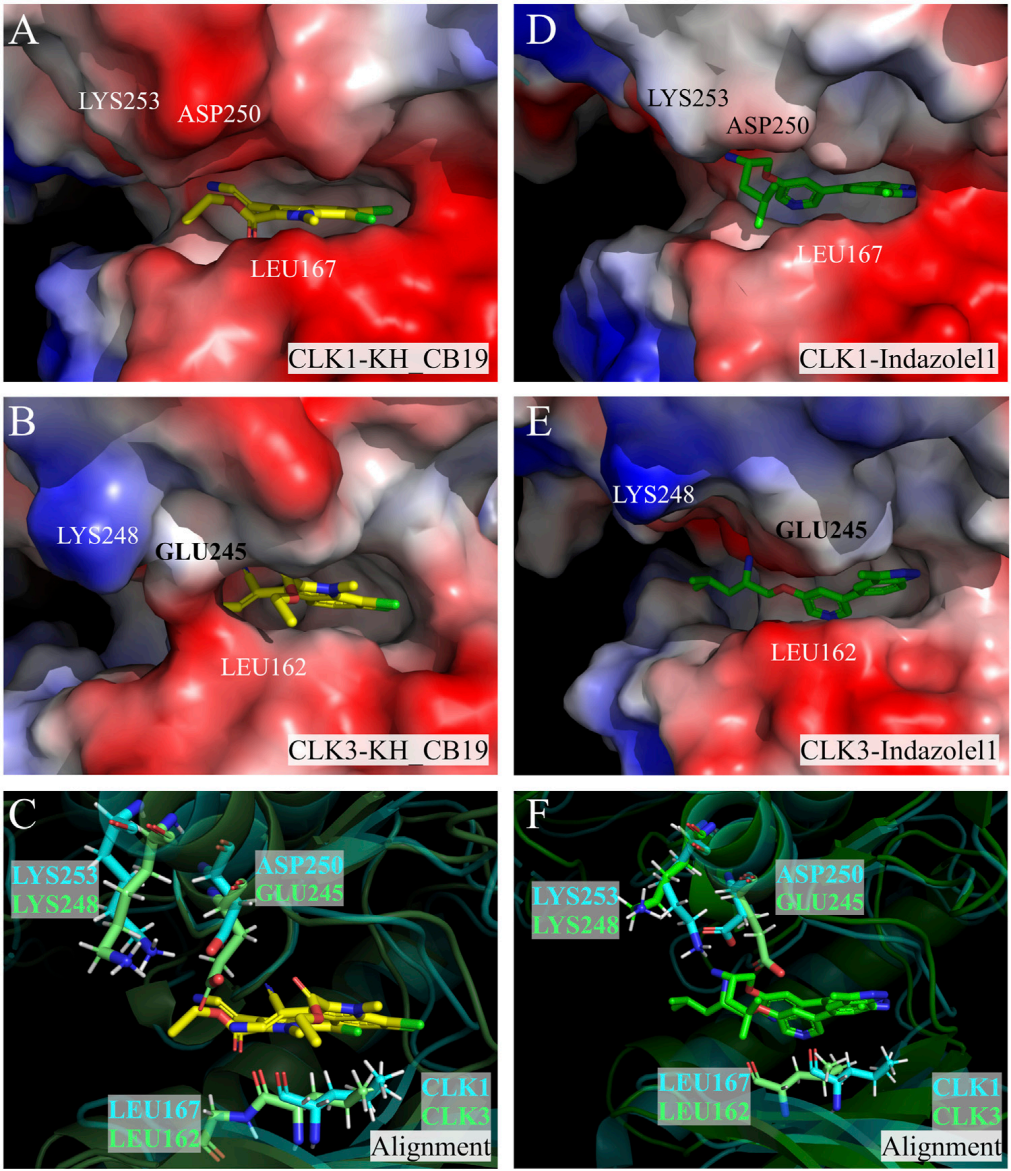
Protein vacuum electrostatics surface. **(A)** CLK1/KH_CB19, **(B)** CLK3/KH_CB19, **(C)** Alignment of CLK1 and CLK3 with KH_CB19 bound, **(D)** CLK1/Indazolel1, **(E)** CLK1/Indazolel1, and **(F)** Alignment of CLK1 and CLK3 with Indazolel1 bound. The blue and red colors represent positive and negative charges of the surface, respectively. The yellow and green molecules in the center of the pocket are KH_CB19 and Indazolel1, respectively. CLK1 is represented with blue ribbon, and CLK3 is represented with green ribbon.

To compare the shape and size between the binding pockets of CLK1 an CLK3, and to reveal the distribution of electrostatic potential of CLK residues, PyMol was used to generate the vacuum electrostatics surface of the CLK complexes (Figure 15).

According to the generated binding pocket surface, the change from CLK1-ASP250 to CLK3-GLU245 causes the entrance of the CLK3 pocket to be narrower than CLK1 by about one carbon-carbon single bond distance, which is reflected in the surface of the CLK3-KH_CB19 complex as the GLU245 in contact with the LEU162 (Figure 15B). Therefore, the longer side chain of amino acid residues may be an obstacle to the entry of the inhibitor, which may be one of the important reasons for the higher inhibitory bioactivity of Indazole1 and KH_CB19 to CLK1 than CLK3.

Furthermore, it has been reported that CLK3 has a protruding LYS248 residue at the entrance of the pocket that alters the surface charge distribution leading to the difficulty of ligand entry (Moyano et al., 2020; Lee et al., 2019), which can indeed be reflected on the surface of PyMol-generated proteins (Figures 15A, B). However, by directly comparing the conformational differences of the above residues (CLK1-LYS253, CLK3-LYS248), the distinctions are very small and not sufficient to impact the change of the surface charge distribution in the corresponding regions (Figure 15C). And the PyMol-generated protein vacuum electrostatics surface may be influenced by short cutoffs, truncations, and lack of solvent screening, and should be viewed with skepticism when observed. Therefore, we have reservations about the effect of CLK3-LYS248 on the biological activity of CLK1/3.

### Hirshfeld surface analysis

**FIGURE 16:**
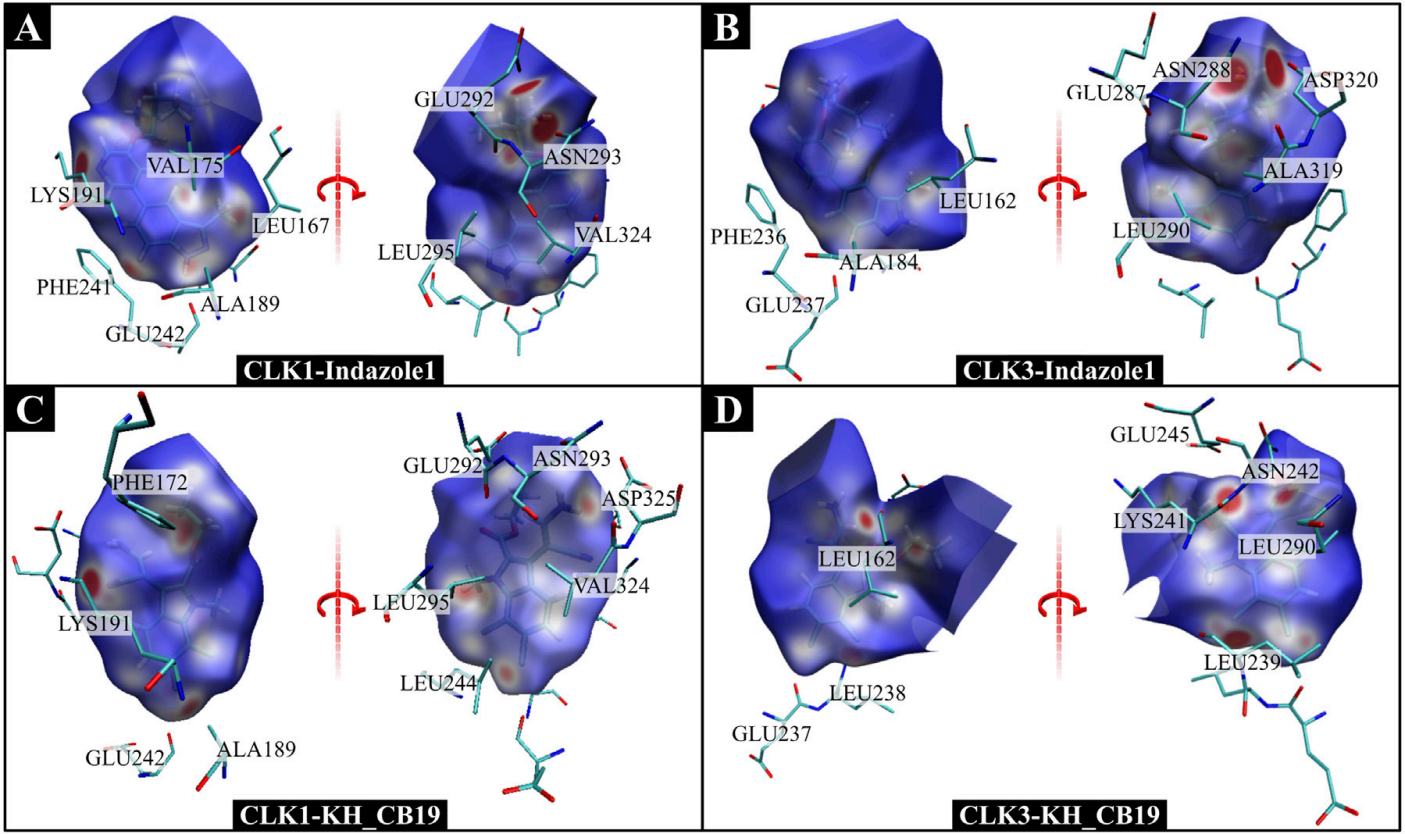
Hirshfeld surface of the binding pocket of the complexes. The variation of electron density from 0.000 to 0.015 a.u. corresponds to the blue-white-red pocket surface color. The Hirshfeld surface of a complex is represented by two diagrams flipped 180°. **(A)** CLK1/Indazolel1, **(B)** CLK3/Indazolel1, **(C)** CLK1/KH_CB19, and **(D)** CLK3/KH_CB19.

Hirshfeld surface analysis is generated by calculating the electron density distribution between atomic fragments. The red part of the Hirshfeld surface is the high electron density region, indicating strong non-covalent interactions, while the white surface indicates weak interactions. As with the results of molecular docking and MD simulations, LYS191 of CLK1 both formed strong interactions with Indazolel1 and KH_CB19, and were shown as red surfaces (Figure 16). The VAL324 isopropyl side chain of CLK1 interacts hydrophobically with both compounds, which shows up as a white surface. The amino group of Indazolel1 forms strong hydrogen-bonding interactions with GLU292 and ASN293 of CLK1, and the main and side chains of GLU292 are involved in the formation of two hydrogen bonds, respectively (Figure 16A). And the amino acid residues of Indazolel1 that interact with CLK3 to form hydrogen bonds changed to ASN288 and ASP320 (Figure 16B). This result supports that Indazolel1 has a different conformation in CLK1/3 and may be directly responsible for the difference in selective inhibitory activity. Compared to Indazolel1, KH_CB19 forms a substantially weaker hydrogen bond interaction with GLU292 and ASN293 of CLK1, which is responsible for its lower inhibitory activity (Figure 16C). Finally, KH_CB19 forms a strong contact with the backbone of ASN242 of CLK3 caused by hydrogen bonding interactions (Figure 16D), which is consistent with the MD simulation results.

## Conclusion

Here, the selectivity mechanism of inhibiting CLK1 over CLK3 was investigated from a computational perspective. Structural comparisons and protein contact pattern analysis revealed structural and pocket differences between CLK1 and CLK3, molecular docking and MM-GBSA binding free energy calculations revealed the interaction patterns of ligand-protein, MD simulations broadened our understanding of the behavior of the complexes in the solvent environment, and QM/MM calculation disclosed the electron density distribution of CLK complexes. It was found that CLK1 and CLK3 have similar spatial structures and highly conserved residue sequences, but the differences still exist for the design of potential selective inhibitors. Due to the increased length of the Glu245 side chain, CLK3 provides a narrower entrance to the binding pocket than CLK1, creating a barrier to entry of the inhibitor. In addition, the presence of a hydrophobic space consisting of a DFG motif within the CLK1 binding pocket plays an important role in the binding of the inhibitor. In contrast, the side chain change of the Ala319 of CLK3 leads to a reduction in its hydrophobic effect and is associated with conformational differences in the inhibitor, being one of the key selective factors. Moreover, the additional hydrogen bonding of the benzo pyrazolyl in Indazole1 in the hydrophobic pocket and the stable hydrogen bonding of the nitrogen atom in its pyridyl in contact with Lys191 guarantee its high inhibitory bioactivity against CLK1, while the corresponding residue Lys186 of CLK3 is difficult to generate effective contact with Indazole1 because of the difference in protein conformation. Therefore, molecules with longer electronegative groups or molecules lacking hydrophobic binding interactions with the interior of the pocket may be able to break the distance limit and establish new hydrogen-bonding interactions with CLK3-Lys186 thereby losing or weakening the selectivity for CLK1. Further, Indazole1 can form more strong and stable hydrogen bonding interactions in the pocket of CLK1 than CLK3. Finally, the positive ionizable area provided by the amino group in Indazole1 and KH_CB19 interacting with Glu292 and Asn293 of CLK1, and the contact of the hydrophobic group with Phe172, are the basis for the stable inhibition of CLK1. This study provides an in-depth analysis of the binding mode of Indazole1 and KH_CB19 to CLK1/3 proteins and the selectivity factors, which is useful information for the rational design of novel and selective inhibitors in the future.

## Data Availability

The data that support the findings of this study are available on request from the corresponding author.
