# Supplementary material for: Stable H-bond networks are crucial for selective CLK1 inhibition: a computational perspective
Source: Front Chem. 2025 Jun 17;13:1582515. doi: 10.3389/fchem.2025.1582515 (PMC12209269; doi:10.3389/fchem.2025.1582515)
Supplement: Supplementary file 1 [file DataSheet1.pdf]

SUPPLEMENTARY INFORMATION FOR

Stable H-bond networks are crucial for selective CLK1 inhibition: a computational perspective

Yuzhou Huang[a,b], Baichun Hu[b], Haihan Liu[b], Jian Wang[b], Na Duan[a*]

a. Department of Cardiology，The People's Hospital of Liaoning Province, Shenyang 110016, People's Republic of China

b. Key Laboratory of Structure-Based Drug Design ＆ Discovery of Ministry of Education, Shenyang Pharmaceutical University, Shenyang 110016, People's Republic of China

Yuzhou Huang and Baichun Hu contributed equally to this work.

Corresponding author: Na Duan, E-mail: naduan@hotmail.com.

This file includes:

Supplementary Figure S1 to Figure S5;

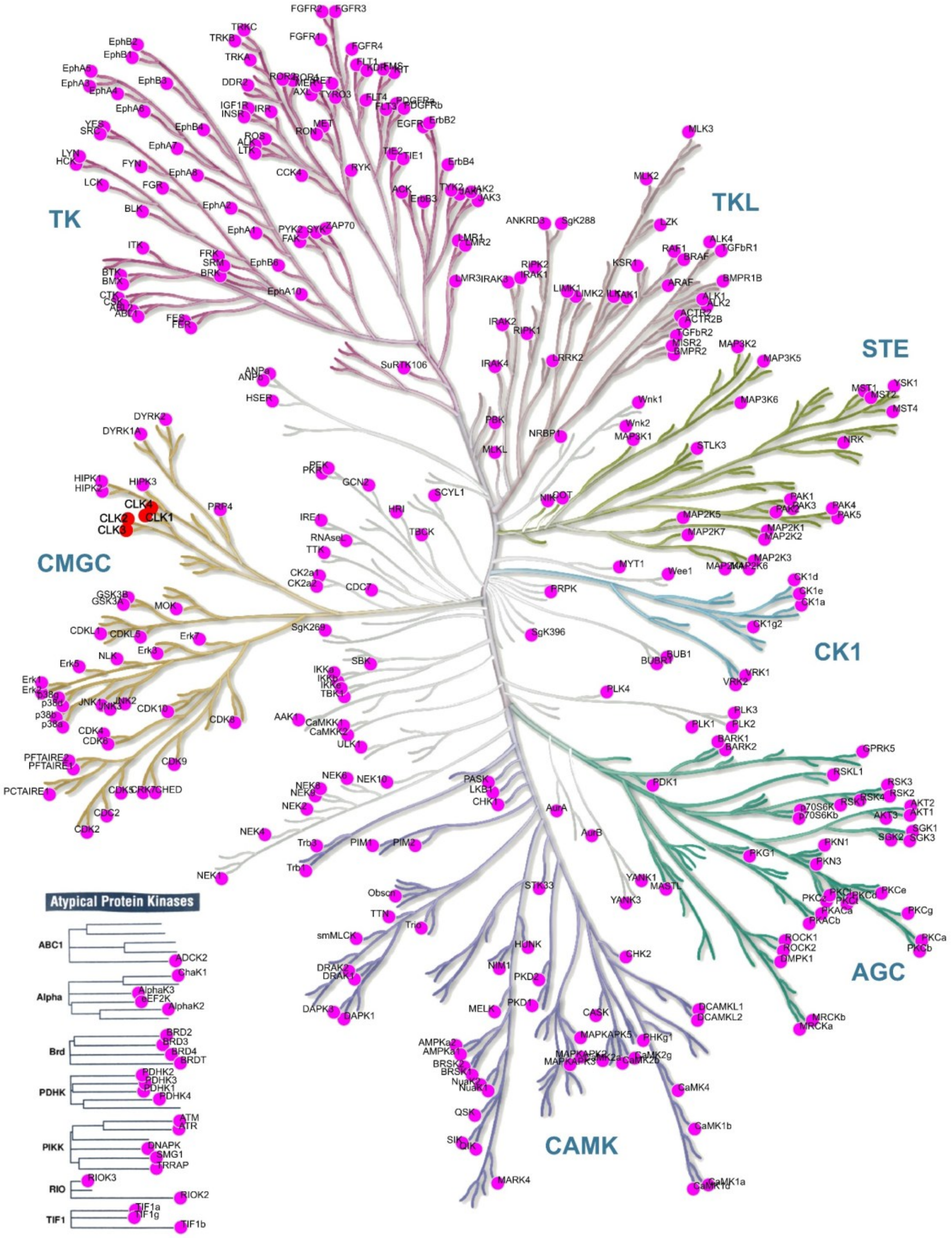

Figure S1. Kinome tree with CLKs marked by red circles and cancer-associated kinases marked by purple circles. Source: KinMap software. Illustration reproduced courtesy of Cell Signaling Technology, Inc. (www.cellsignal.com).

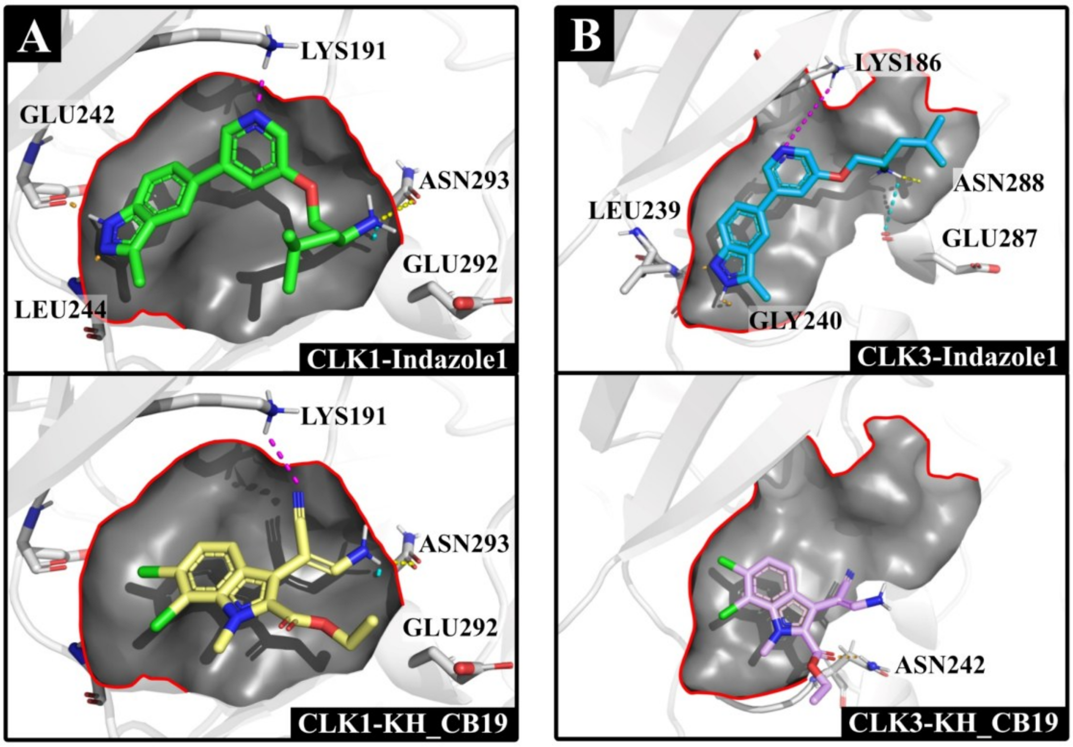

Figure S2. Hydrogen bonding modes of the complexes within CLK1 or CLK3 pocket. (A) Indazolel1 (green stick) and KH_CB19 (yellow stick) binds within the CLK1 pocket. (B) Indazolel1 (blue stick) and KH_CB19 (purple stick) binds within the CLK3 pocket. The hydrogen-bonding interactions with different residues are indicated by dashed lines of different colors, respectively.

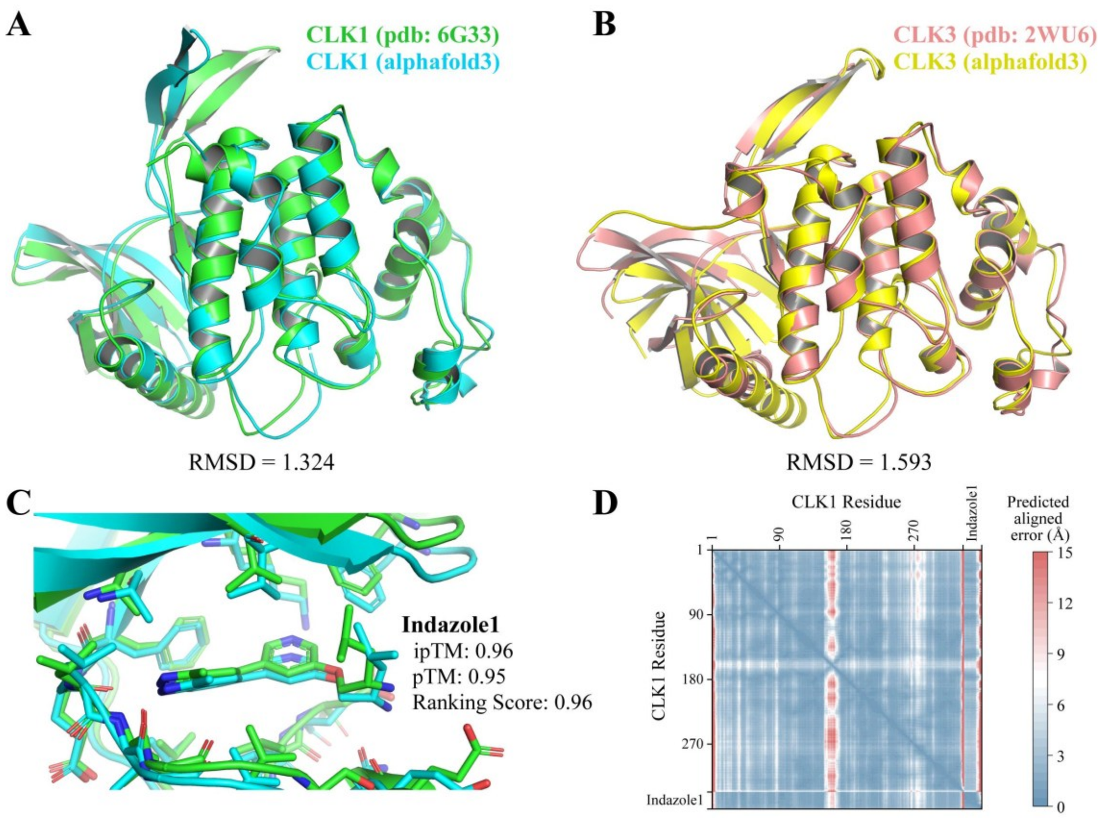

Figure S3. Comparison between AlphaFold3 predicted structures and experimental structures. The RMSD value was calculated using pymol. (A-B) Overall structure alignment. (C) The conformation of the Indazole1 in the experimental and predicted structures is consistent with high ipTM (interface predicted TM-score) and pTM (predicted TM-score). (D) The high ipTM value is reflected in the predicted aligned error (PAE) plot.

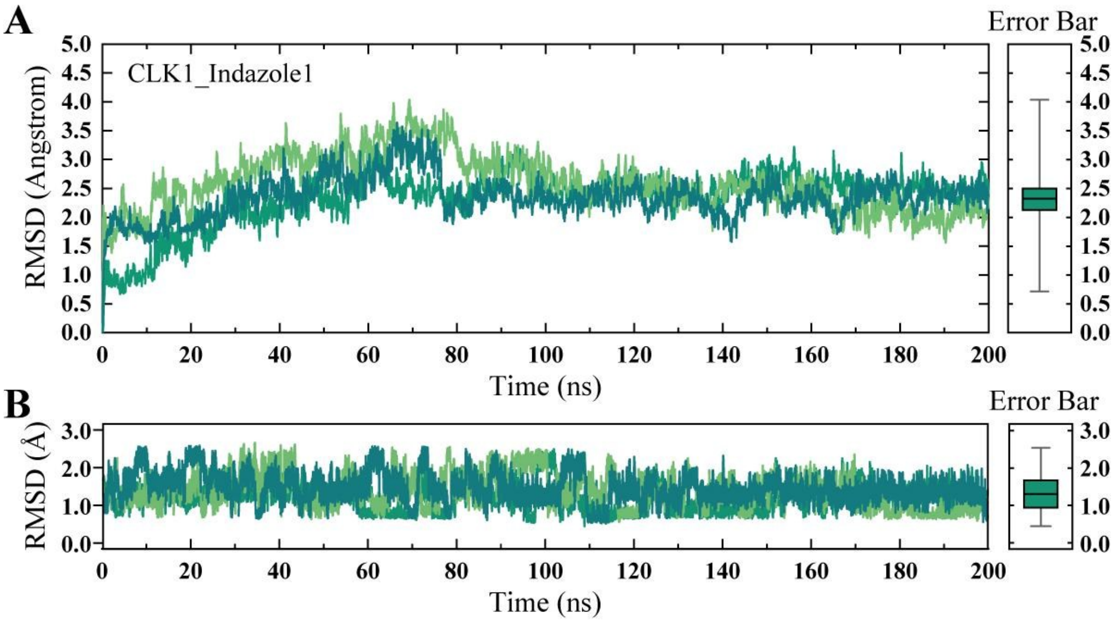

Figure S4. RMSD value of 200 ns MD simulation for CLK1_Indazole1 complex. (A) Protein RMSD. (B) Ligand RMSD. The three sets of repeated simulations are represented by lines of diffrent colors. The box-plot graph on the right side indicates the average value within 100 ns of simulations. The box extends from the 25th to 75th percentiles. The line in the middle of the box is plotted at the median. Error bar indicated the min-max value of RMSD during the simulations.

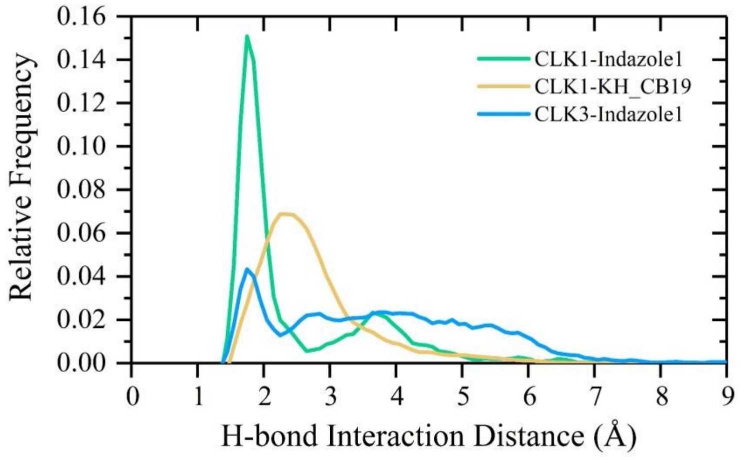

Figure S5. Relative frequency distribution of the distances of key hydrogen-bond interactions

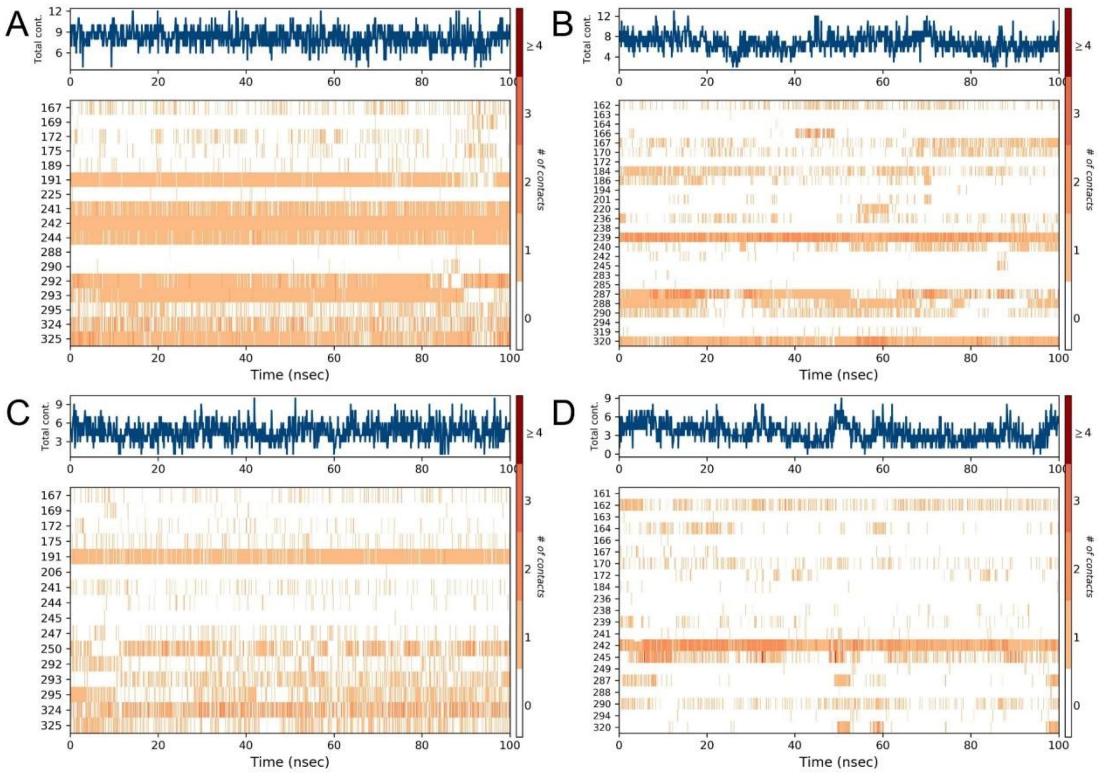

Figure S6. The protein-ligand contacts over the entire simulations. (A) CLK1/ Indazolel1. (B) CLK3/ Indazolel1. (C) CLK1/ KH_CB19. (D) CLK3/ KH_CB19. The bottom panel shows the total number of specific contacts the protein makes with the ligand over the course of the trajectory. The upper panel shows which residues interact with the ligand in each trajectory frame. Some residues make more than one specific contact with the ligand, which is represented by a darker shade of orange, according to the scale to the top of the plot.

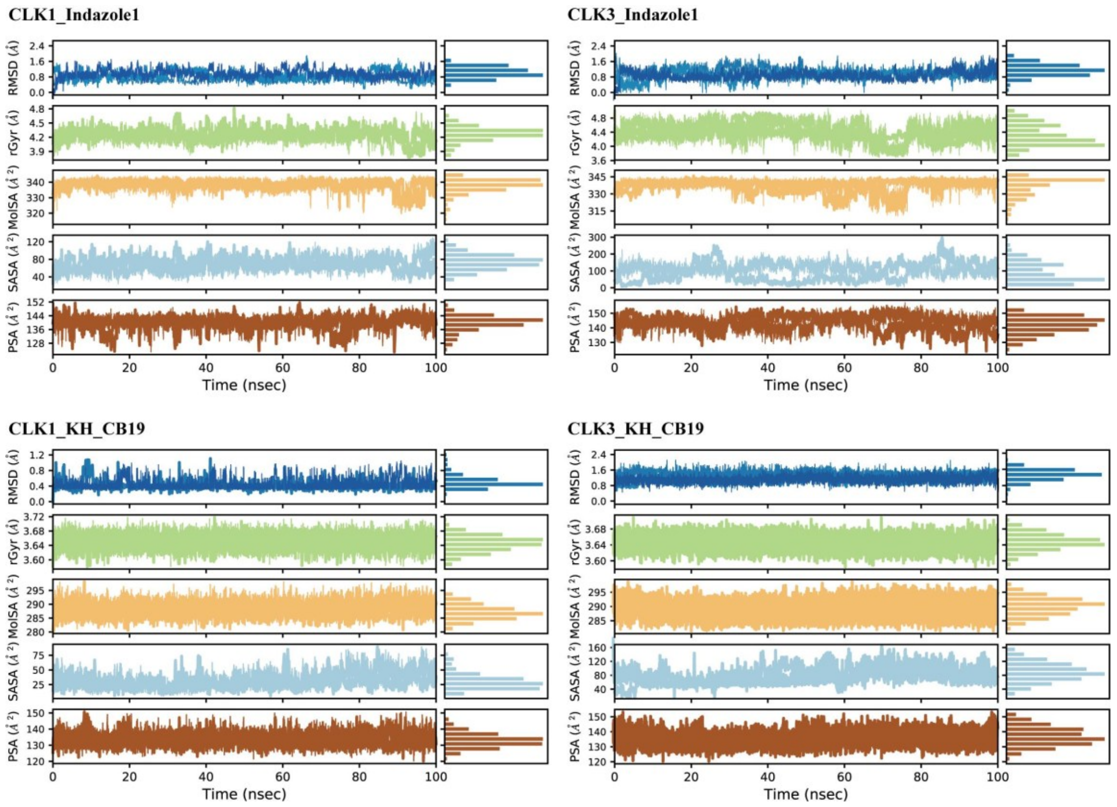

Figure S7. Variation properties of hits during 100 ns simulations. Ligand RMSD: Root-Mean-Square deviation of the ligand relative to the reference conformation. The three sets of repeated simulations are represented by lines of similar colors. The radius of Gyration (rGyr): The "extendedness" of the ligand is equal to its principal moment of inertia. Intramolecular Hydrogen Bonds (intraHB): The number of hydrogen bonds in the ligand molecule. Molecular Surface Area (MolSA): Molecular surface’s calculation, which is equal to van der Waals surface area. Solvent Accessible Surface Area (SASA): The surface area of molecules that water molecules can contact. Polar Surface Area (PSA): Solvent accessible surface area contributed by oxygen and nitrogen atoms.
